# The role of chromatin accessibility in directing the widespread, overlapping patterns of *Drosophila *transcription factor binding

**DOI:** 10.1186/gb-2011-12-4-r34

**Published:** 2011-04-07

**Authors:** Xiao-Yong Li, Sean Thomas, Peter J Sabo, Michael B Eisen, John A Stamatoyannopoulos, Mark D Biggin

**Affiliations:** 1 Genomics Division, Lawrence Berkeley National Laboratory, 1 Cyclotron Road MS 84-171, Berkeley, CA 94720, USA; 2 Howard Hughes Medical Institute, University of California Berkeley, 176 Stanley Hall #3220, Berkeley, CA 94720, USA; 3 Department of Genome Sciences, University of Washington, Foege S310A, 1705 NE Pacific Street, Box 355065, Seattle, WA 98195, USA; 4 Department of Molecular and Cell Biology, University of California Berkeley, 176 Stanley Hall #3220, Berkeley, CA 94720, USA

## Abstract

**Background:**

In *Drosophila *embryos, many biochemically and functionally unrelated transcription factors bind quantitatively to highly overlapping sets of genomic regions, with much of the lowest levels of binding being incidental, non-functional interactions on DNA. The primary biochemical mechanisms that drive these genome-wide occupancy patterns have yet to be established.

**Results:**

Here we use data resulting from the DNaseI digestion of isolated embryo nuclei to provide a biophysical measure of the degree to which proteins can access different regions of the genome. We show that the *in vivo *binding patterns of 21 developmental regulators are quantitatively correlated with DNA accessibility in chromatin. Furthermore, we find that levels of factor occupancy *in vivo *correlate much more with the degree of chromatin accessibility than with occupancy predicted from *in vitro *affinity measurements using purified protein and naked DNA. Within accessible regions, however, the intrinsic affinity of the factor for DNA does play a role in determining net occupancy, with even weak affinity recognition sites contributing. Finally, we show that programmed changes in chromatin accessibility between different developmental stages correlate with quantitative alterations in factor binding.

**Conclusions:**

Based on these and other results, we propose a general mechanism to explain the widespread, overlapping DNA binding by animal transcription factors. In this view, transcription factors are expressed at sufficiently high concentrations in cells such that they can occupy their recognition sequences in highly accessible chromatin without the aid of physical cooperative interactions with other proteins, leading to highly overlapping, graded binding of unrelated factors.

## Background

*In vivo *crosslinking studies show that a wide range of animal transcription factors each bind to many thousands of DNA regions throughout the genome and that not all of this binding is necessarily functional (for example, [1-19]). For example, our studies of over 20 transcriptional regulators in the *Drosophila *blastoderm embryo show that the few hundred most highly bound DNA regions include all of these proteins' known target *cis*-regulatory modules (CRMs) and are preferentially associated with developmental control genes and genes whose expression is strongly patterned in the blastoderm [1-3,14,17,19]. In contrast, the thousands of more poorly bound regions are preferentially associated with genes not transcribed in the early embryo and/or housekeeping genes, and are frequently present in poorly conserved non-coding DNA or in protein coding sequences. In addition, there is a surprisingly high overlap in the genomic regions bound by biochemically and functionally unrelated animal transcription factors *in vivo *[3,17,20], with the distinct biological specificities of factors being determined by quantitative differences in their occupancy on these shared regions [3,17,21,22].

What biochemical mechanisms could be responsible for these widespread, overlapping patterns of animal factor binding? Most animal transcriptional regulators recognize short degenerate DNA sequences that occur frequently near most genes [23]. Only a subset of these sites, however, are highly occupied *in vivo *in a given cellular or developmental context, and the level of occupancy at each site correlates only poorly with a given factor's intrinsic DNA recognition properties [3,6,14,24,25]. Thus, as long recognized, one or more mechanisms must differentially alter the relative occupancy of factors across the genome.

Two such mechanisms have been characterized. The first is direct heteromeric cooperative interactions between pairs of factors bound to adjacent sites in the genome that selectively increase occupancy only to regions where appropriately spaced sites for both factors occur [26-30]. The second is competition for DNA binding with other sequence-specific factors, nucleosomes or other chromatin-associated proteins that selectively reduces binding at a subset of sites [31-39]. While there is evidence that both have some influence on DNA binding *in vivo *[12,25,26,30-32,38-45], there has been no systematic effort to quantify the relative contributions of these positive and negative effects on the overall pattern of factor binding.

One common set of models invokes a prominent role for direct cooperative interactions, suggesting that transcription factors cannot significantly occupy their functional target sites without such interactions between factors [26-30]. These 'direct cooperativity' models have been used to predict that transcription factors will bind highly selectively in non-overlapping patterns, each factor binding to relatively few genes [28,29], and that factors with similar intrinsic DNA recognition properties, such as the HOX proteins, may be targeted to different genes through differential interactions with cooperativity partners [26,30]. These predictions, however, are difficult to reconcile with the measured patterns of DNA binding *in vivo *and, in the case of HOX factors, with their ultimate regulation of a very large pool of common genes [2,46].

Instead, to explain the widespread, overlapping patterns of factor binding in animals, we have previously suggested that transcription factors are expressed at sufficiently high cellular concentrations that they detectably occupy most high and moderate affinity recognition sequences that are physically accessible in the context of chromatin, without the aid of heteromeric cooperative interactions with other factors [3,14,41,46]. In this 'widespread binding' model, nucleosomes and other chromatin proteins would block access to much of the genome [12,25,31,32,40-45]. At the same time, accessible, nucleosome-depleted regions, such as active CRMs, would be bound at high levels by factors exerting an essential function, but would also be bound at lower levels by other factors interacting opportunistically with fortuitously occurring cognate recognition sequences.

Here we seek to quantify the relative contributions of the direct cooperativity and widespread DNA binding models in the context of the quantitative genome-wide *in vivo *binding patterns of *Drosophila *developmental regulators. Genome wide DNaseI digestion data are used to provide a biophysical measurement of the access an exogenous protein has to DNA in nuclei. Since the access a protein has to DNA must affect its level of occupancy on DNA, the DNaseI data measure the contribution to the final pattern of factor binding due to competitive inhibition of binding. In contrast, local genome accessibility is not altered, *per se*, by direct heteromeric cooperative interactions. Thus, by establishing the quantitative correlation between accessibility and levels of factor binding, we can both determine accessibility's contribution to DNA binding and set an upper limit, by the extent of non-correlation, for the contribution that direct heteromeric cooperativity makes.

It is important to note that indirect cooperativity, a mechanism by which binding of two or more factors mutually increase each others ability to competitively displace a nucleosome without making direct physical contacts with each other [47-56], is quite distinct from direct cooperativity. Indirect cooperativity is fully consistent with the widespread binding model. It assumes that at least some factors are expressed at sufficiently high concentrations that they can bind their sites without direct interactions with other factors. It also provides a ready explanation for the high overlap in factor binding because it naturally leads to increased binding of any factors whose recognition sites lie within the DNA region from which a nucleosome has been displaced. Here, however, we make no attempt to distinguish whether this or other mechanisms are the chief cause of the differential accessibility of the genome. By using direct independent measurements of accessibility and then by considering the effect this has on each factor separately, we unlink targeting of individual factors from the challenging question of how the hundreds of transcription factors expressed in each cell, together with the chromatin remodeling/modification enzymes that they recruit, alter chromatin structure [34,35,37-40,57,58].

## Results

### Factor binding is concentrated in highly accessible chromatin

The accessibility of genomic DNA sequences in the context of chromatin *in vivo *has classically been studied using digestion of DNA in isolated nuclei by the non-specific endonuclease DNaseI [59-61]. Using a high-throughput version of this assay (DNase-seq) [62,63], we have previously profiled DNA accessibility genome-wide in native chromatin at high resolution across stages 5, 9, 10, 11 and 14 of *Drosophila *embryogenesis, spanning the first 11 hours of development (S Thomas *et al*., submitted). Even though data for independent replicas from collections of embryos at the same stage of development were highly reproducible (r ≥ 0.91; S Thomas *et al*., submitted; Additional files 1 and 2), to derive a conservative picture of chromatin accessibility, and to minimize the effect of experimental variability, we reanalyzed these data to identify genomic regions with increased DNaseI sensitivity at a 5% false discovery rate (FDR) that were concordant between pairs of replicas. We identified between 16,217 and 24,373 such DNaseI accessible regions per stage, collectively spanning 9 to 13% of the euchromatic genome (Additional files 1, 2 and 3). Consistent with our original results (S Thomas *et al*., submitted), approximately half of the accessible regions present at a particular stage show little change in accessibility over time, whereas the remaining regions display marked increases or decreases in DNaseI sensitivity during embryogenesis.

We next compared the DNase-seq data for stage 5 embryos to *in vivo *DNA binding data at the same stage. At this point in development, the embryo is a single layer of approximately 6,000 undifferentiated cells, which are each likely to have similar patterns of chromatin structure, providing a relatively simple system for our analysis [64]. We used DNA binding data for 21 sequence-specific transcription factors, TFIIB, and the transcriptionally active form of RNA polymerase II that had been quantified by genome-wide *in vivo *formaldehyde crosslinking (ChIP-chip) [14,17]. Only high-confidence bound regions above the 1% FDR threshold were examined, giving a conservative picture of the total amount of factor binding.

An extensive set of controls indicate that our ChIP-chip data provide an accurate measure of the relative levels of factor directly contacting the different genomic DNA regions to which they are crosslinked [3,14,17,41,65]. For example, *in vitro *controls show that formaldehyde crosslinking of purified transcription factors to naked DNA is proportional to factor occupancy on the DNA; quantitative PCR and bacterial artificial chromosome 'spike-in' experiments show that the whole genome amplification used in our ChIP-chip experiments preserves the relative differences in enrichment of various genomic regions; and *in vivo *UV crosslinking results show that similar data are obtained when protein-protein crosslinking is absent. In light of a recent paper showing that sonication of intact nuclei can lead to the preferential release of short (<350 bp) DNA fragments from accessible genomic regions [66], we also note that the crosslinked DNA used in our ChIP-chip experiments is sonicated only after it has been purified away from non-covalently attached proteins and that the resulting DNA fragments are mostly longer than 350 bp (mean size approximately 600 bp). As a result, our crosslinked input DNA samples show no evidence of bias towards genomic regions that are either highly accessible to DNaseI digestion or highly bound by factors (Additional file 4). Further, the quantification of ChIP-chip data (ChIP-chip scores) used throughout this and our previous work, with the exception of that in Additional file 4, were calculated by dividing the array hybridization signal from a factor immunoprecipitation by the array signal from the exactly matched, 'input' crosslinked DNA sample [14], which would correct for any DNA extraction bias that had occurred.

**Figure 1:**
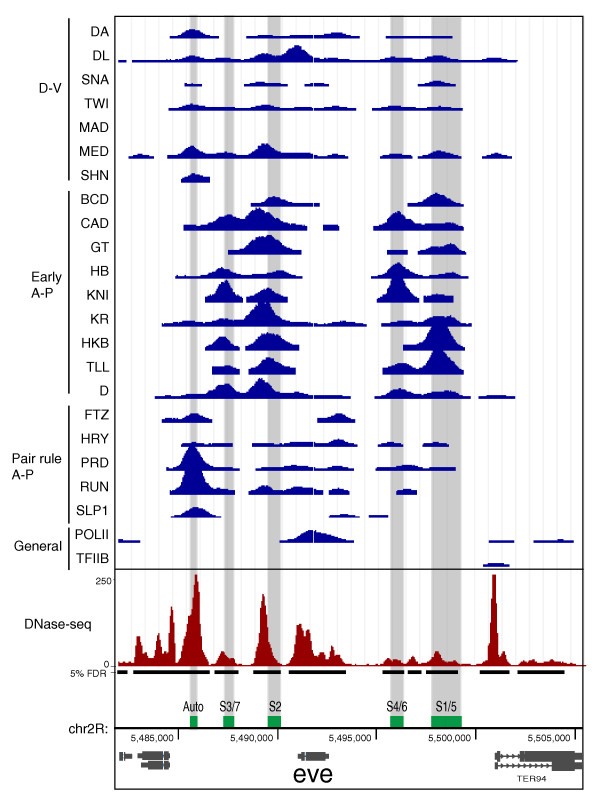
**DNaseI accessibility and *in vivo *DNA binding by transcription factors across the *eve *locus**. DNA binding in stage 5 embryos is shown as ChIP-chip scores (blue) for 675-bp windows that fall above a 1% FDR threshold for 21 sequence-specific transcription factors, TFIIB and the transcriptionally active form of RNA polymerase II (POLII). The sequence-specific factors are grouped into three major regulatory classes that regulate patterning along the Dorsal-Ventral axis of the embryo (D-V), initiate patterning along the Anterior-Posterior axis (Early A-P), or establish later pair rule patterns along the Anterior-Posterior axis (Pair rule A-P). DNaseI accessibility at stage 5 is shown for 75-bp windows of sequence tag density (red) along with the locations of accessible regions above the 5% FDR threshold (black bars). At the bottom, the locations of major RNA transcripts are shown (grey) as well as the autoregulatory CRM (Auto) and the four stripe initiation CRMs (S3/7, S2, S4/6 and S1/5) (green). Nucleotide coordinates in the genome are given in base pairs.

**Figure 2:**
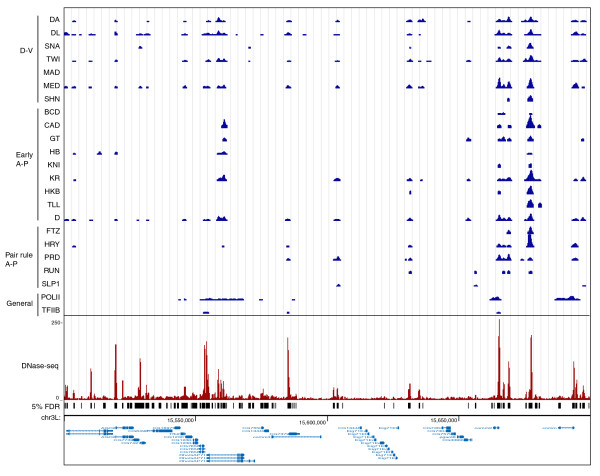
**DNaseI accessibility and *in vivo *DNA binding by transcription factors across a 200-kb genomic region**. The figure is labeled using the same conventions in Figure 1 except that the RNA transcript locations are shown in light blue at the bottom.

Figure 1 compares DNase-seq and the ChIP-chip data for the *even-skipped *(*eve*) locus at stage 5. This well characterized target gene contains five CRMs that molecular genetics indicate are each bound and regulated by combinations of the 21 regulatory factors at this stage of embryo development [67-69]. These proteins are expressed in different spatial patterns and either activate or repress transcription such that, while the *eve *gene is only expressed in a subset of cells, each CRM is expected to be accessible and bound by at least some of these factors in all cells [67-69]. Consistent with this, all five CRMs show peaks of DNA binding for many of the 21 factors (Figure 1). Local peaks of DNaseI accessibility align very well with both the CRMs and peaks of factor binding, with the DNase-seq peaks varying in intensity (reflected in the density of mapped DNA sequence tags) over approximately a ten-fold range (Figure 1). While this variation in peak intensity is higher than that expected and may reflect differences in experimental bias in each assay, analyses presented later in the paper indicate that, when averaged over multiple regions, DNase-seq signals do correlate with levels of factor occupancy. A high overlap between genomic regions identified by DNase-seq and ChIP-chip is also apparent across much longer regions of the genome (Figure 2), wherein the strongest peaks of factor binding almost uniformly align with major peaks of DNaseI accessibility in stage 5 chromatin.

To quantify the global correlation between factor binding and DNaseI accessibility, we first determined the proportion of ChIP-chip peak regions that overlapped 5% FDR accessible regions at stage 5 (see Materials and methods; Additional file 5). Combining data from all 21 factors, RNA polymerase II and TFIIB, we observed a strikingly high overlap (mean 87%, range 71 to 99%, probability of observing a higher overlap randomly <1 × 10^-16^). We also determined the proportion of accessible regions that coincided with genomic regions bound by one or more of the 21 sequence-specific factors. Although stage 5 DNaseI accessible regions encompass only approximately 12% of the euchromatic genome, 61% of these regions coincide with binding for at least one of the 21 factors (probability of a greater overlap occurring by chance <1 × 10^-16^), or 65% if RNA polymerase and TFIIB binding are included. By contrast, only 7% of the genome that is at least 500 bp away from accessible chromatin is covered by 1% FDR ChIP-chip regions (probability of getting less overlap at random <1 × 10^-16^). Moreover, the most accessible regions displayed even higher levels of overlap with regulatory factor binding sites. Of the 5,000 most accessible regions, 95% are occupied by at least one of the 21 factors above the 1% FDR threshold, with nearly monotonically decreasing overlap with decreasing chromatin accessibility (Additional file 6).

### Quantitative relationship between genome accessibility and factor occupancy

**Figure 3:**
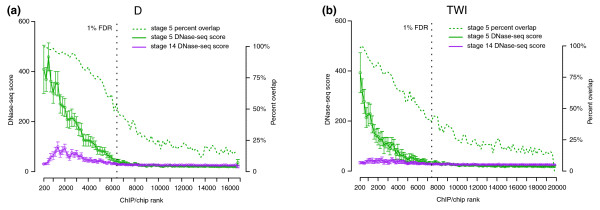
**Levels of factor occupancy and genome accessibility correlate**. The median DNase-seq tag density in non-overlapping cohorts of 200 1-kb ChIP-chip peaks is shown down the ChIP-chip rank list (continuous lines). The ChIP-chip data are from stage 5 embryos and the DNaseI accessibility data are from stages 5 (green) and 14 (purple). The 95% confidence limit for median DNaseI accessibility of each cohort is indicated. Shown also is the percent of ChIP-chip peaks that are overlapped by 5% FDR accessible regions in stage 5 embryos (dashed green line). The regions most highly bound by transcription factors are to the left along the x-axis and results are plotted as far as the 25% FDR cutoff. The location of the ChIP-chip 1% FDR threshold is indicated by the vertical black dotted line. Results for the regulatory transcription factors **(a) **Dichaete (D) and **(b) **Twist (TWI) are shown. Additional file 7 shows plots for all 21 regulators.

Because our previous studies establish that it is the level of regulatory factor occupancy on a given genomic region that is an important determinant of function, rather than if a region is detectably bound or not [2,3,14,17], we next performed a quantitative comparison of factor binding and accessibility. We calculated median DNaseI scores for cohorts of 200 ChIP-chip peaks, grouped and ranked according to their ChIP-chip scores in stage 5 embryos (see Materials and methods). This analysis revealed that, for each factor, the regions that are most highly bound are significantly more accessible than regions bound at lower levels (Figure 3; Additional file 7). This result is most compelling for those factors with the most regions identified above the 1% FDR ChIP-chip threshold, since in these cases false positives should not contribute significantly to the median DNaseI score above this threshold; notably, however, all factors show this trend.

We confirmed that the aforementioned relationship is quantitative - that is, that the lower median accessibility of cohorts of poorly bound regions largely derives from reduced accessibility of each region rather than a reduced number of accessible regions versus highly bound cohorts. This is illustrated clearly by the fact that the proportion of ChIP-chip peaks that overlap accessible regions reduces more gradually down the rank list than do DNaseI scores (Figure 3; Additional file 7). For example, for the sequence-specific factor Dichaete (D) at ChIP-chip rank 2,000 when accessibility is reduced by two-fold, the percent overlap drops only marginally.

The plots in Figure 3 also show that regions bound highly by factors in stage 5 are much less accessible at stage 14 than at stage 5, even though we have previously shown that both stages contain a similar number and length of accessible regions, and the median accessibility of accessible regions at stage 14 is fully 78% of that at stage 5 (S Thomas *et al*., submitted; Additional file 2). Thus, most genomic regions bound at high levels by regulatory factors at stage 5 have their accessibility specifically reduced at later stages of development, consistent with the known inactivation of many early active CRMs.

### Genome accessibility and intrinsic factor specificity determine occupancy *in vivo*

The above analyses establish a close quantitative relationship between genome accessibility and local levels of factor binding. They do not, however, establish whether the pattern of binding is determined principally by genome accessibility *per se*, or whether it is the binding of regulatory factors that potentiates chromatin accessibility. As described in the Introduction, ultimately, it is the combined action of all of the hundreds of sequence-specific factors in a given cell, together with the chromatin remodeling proteins that they recruit, that is likely to determine the pattern of chromatin accessibility [34,35,37-40,47-58]. We therefore focused our attention on the more immediately tractable question of whether, for each single factor in turn, observed chromatin accessibility (however originated mechanistically) has a major effect on determining that factor's binding pattern.

To address this question, we first compared the influence on levels of *in vivo *factor occupancy of both genome accessibility and the intrinsic specificity of factors for naked DNA as determined *in vitro *using purified protein. All of the 16 factors for which there are sufficiently accurate position weight matrices (PWMs) of intrinsic specificity [17,70] (Berkeley *Drosophila *Transcription Network Project (BDTNP), unpublished data) were examined. We segmented the genome into accessible and closed chromatin compartments based on the 5% FDR accessible regions. We then scanned each compartment and annotated all significant matches to each of the 16 factor PWMs, and then classified these into several affinity cohorts. To provide a negative control, we also separately identified for each factor equivalent cohorts of matches to sets of PWMs for which the order of nucleotide positions had been randomly permutated. At the location of each match to the genuine or scrambled PWMs, the median ChIP-chip score of the region ±250 bp around the match was calculated. The highest affinity cohorts typically contained 1,000 recognition site occurrences in accessible chromatin and 12,000 in closed regions, whereas the lowest affinity cohorts contained 0.8 and 6.6 million in these regions (Table 1).

**Table 1:** Frequency of DNA affinity cohort recognition sequences in accessible and closed genome regions

Affinity cohort	*P*-values included	Mean number of PWM matches for factors in 5% FDR accessible regions	Mean number of PWM matches for factors in closed genomic regions
-5	*P *<1e-4.5	1,145	12,344
-4	1e-3.5 > *P *> 1e-4.5	9,938	96,853
-3	1e-2.5 > *P *> 1e-3.5	94,126	825,406
-2	1e-1.5 > *P *> 1e-2.5	811,773	6,596,274

**Figure 4:**
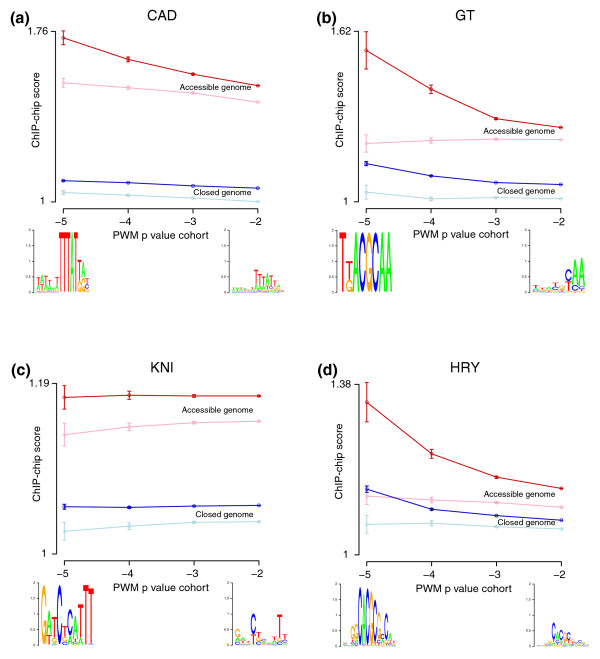
**Factor recognition sites in DNaseI accessible regions are more highly bound *in vivo *than sites in closed chromatin**. Separately for each transcription factor, all significant recognition sequences in the euchromatic genome for four affinity cohorts were identified using PWMs derived from *in vitro *DNA binding data (Table 1) [17,70]. In addition, matches to ten PWMs derived by random permutation of nucleotide position order were derived for each factor. Sites in each affinity cohort for both the genuine and scrambled PWMs present were each classified as either accessible or inaccessible, using the 5% FDR DNaseI accessible regions to define accessible regions (Table 1). The median ChIP-chip scores (y-axis) for the 500-bp regions ±250 bp around recognition sites in each affinity cohort were plotted separately for accessible (red lines) and inaccessible (blue lines) genomic regions. Dark red and blue lines show results for the genuine factor PWMs, light red and blue lines the median result for the scrambled PWMs. The highest affinity cohort is to the left (x-axis). Web logo representations of the PWM representing the highest and lowest affinity cohorts of genuine recognition sites are shown at the bottom. The 95% confidence limits for the median ChIP-chip scores are indicated. Plots for **(a) **CAD, **(b) **GT, **(c) **KNI, and **(d) **HRY are shown. Additional file 8 provides similar plots for all 16 factors for which sufficiently accurate PWMs are available.

This analysis revealed that, among genomic regions that contain genuine factor recognition sequences of similar affinity, those in the accessible chromatin (dark red lines in Figure 4 and Additional file 8) are clearly bound at significantly higher levels *in vivo *than those in inaccessible chromatin regions (dark blue lines in Figure 4 and Additional file 8). The fact that the same pattern is evident for 16 factors with widely varying DNA binding specificities (Additional file 8) strongly suggests that the observed correlation is not the result of any sequence bias in regions detected by the DNase-seq assay, but instead reflects genuinely different properties of accessible and closed chromatin regions. Additionally, the fact that such large effects are seen when averaged over thousands to millions of genomic regions strongly suggests that accessibility has a major influence on *in vivo *occupancy genome-wide.

Further, in 13 out of 16 cases (excepting KNI, PRD, and FTZ), genomic regions with higher intrinsic affinity recognition sequences have higher ChIP-chip scores. Even moderate affinity sites, though, appear to mediate DNA binding *in vivo*, albeit at a lower level, as these are occupied at higher levels than matches to scrambled PWMs of equivalent affinity for all 16 factors (compare the dark red and light red lines in Figure 4 and Additional file 8). Thus, both the intrinsic affinity of a factor for a given DNA sequence and the accessibility of the site contribute to the pattern of genome binding *in vivo*.

**Figure 5:**
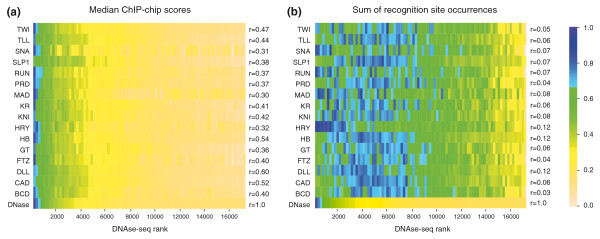
**Accessibility better explains *in vivo *occupancy than does intrinsic affinity**. We identified and grouped 150-bp local peaks of accessibility within DNaseI accessible regions into non-overlapping cohorts of 200 peaks down the DNase-seq rank list. **(a) **The median ChIP-chip score in each cohort for each factor. **(b) **The sum of occurrences of recognition sequences that match the factor's PWM (*P *< 0.003) in each cohort for each factor. The bottom row in each panel shows the relative DNase-seq scores for each cohort. Data for each factor were normalized by scaling the median value for each row and plotted as a heat map. The correlation coefficients of the data for each factor with the DNase-seq scores are shown on the right. The correlations are calculated using data for each accessible region, not the cohort average values.

We next focused exclusively on accessible genomic regions, and asked which component - measured factor occupancy *in vivo *or the intrinsic affinity of factors for DNA - was more closely correlated with chromatin accessibility. To address this, we grouped accessible regions into ranked cohorts of 200 based on the peak density of mapped DNaseI cleavages within each region, and plotted the median ChIP-chip scores and the number of recognition sequences (at the *P *< 0.003 matching level) in each cohort (Figure 5).

For all 16 factors, we found that observed levels of *in vivo *occupancy decline sharply in parallel with accessibility, most strikingly across the few thousand most accessible regions, and more gradually after that over the remaining regions. The fact that a wide array of regulatory factors with markedly different intrinsic DNA binding and biological specificities all show a similar correlation in their levels of occupancy across a diverse array of genomic elements alone implies that some common principle is directing the pattern of binding. The strong correlation of binding with accessibility suggests that the degree of access that factors have to DNA is the common force driving the otherwise surprisingly similar behavior of factors. This view is further supported by the fact that the intrinsic DNA recognition properties of factors correlate much more poorly with accessibility than does *in vivo *occupancy, suggesting that access to DNA plays a larger role in determining occupancy *in vivo *than does intrinsic specificity (r = 0.03 to 0.12 versus r = 0.32 to 0.6; Figure 5). For each factor, the density of recognition sequences drops more gradually down the rank list of accessible genomic regions than do either levels of *in vivo *occupancy or DNase-seq scores (Figure 5). Indeed, for many factors the most accessible cohorts have fewer recognition sites than regions 2,000 to 6,000 down the rank list. There is higher correlation between site density and accessibility for a few factors (especially HRY, RUNT and SNA), which could suggest that these proteins play a pioneering role in determining the pattern of genome accessibility, similar to transcription factors such as the glucocorticoid receptor [44,49]. This correlation, however, is still low (<0.13), suggesting that accessibility is affecting their binding more than any of them are affecting it.

### Developmental alterations in genome accessibility direct changes in factor binding

The above analyses strongly support the 'widespread binding' model in that they suggest that the accessibility of DNA in chromatin plays a major role in determining the pattern of *in vivo *DNA binding for each transcription factor. These analyses, however, are largely of events at a single stage (stage 5). As described above, we have shown that many regions bound by developmental regulators at this stage become inaccessible in later embryogenesis (Figure 3; Additional file 7) and regions bound by factors in later stages are inaccessible at stage 5 (S Thomas *et al*., submitted). Such perturbations of the chromatin landscape during development provide a unique and rigorous opportunity to assess the extent to which the patterns of regulatory factor DNA binding are caused by accessibility, as follows. Since changes in factor binding between stages are necessarily measured on the same genomic regions, any alteration in occupancy cannot be due to differences in DNA sequence, but must instead derive from temporal changes in the influence of other proteins on binding, including occlusion by nucleosomes. While direct positive cooperative interactions with other sequence-specific factors could, in principle, be responsible for most of the temporal alterations in DNA binding, this cannot be the case if these alterations in DNA binding are highly correlated with changed DNA accessibility. In such cases, since changed accessibility must affect factor DNA binding and do so in proportion to the degree of that change, any additional influences on DNA binding due to heteromeric cooperative interactions and other effects must be limited, at most, to the residual extent that altered DNA binding and accessibility do not correlate. In other words, a temporal analysis sets an upper bound on all other influences on factor binding, beyond chromatin accessibility and the intrinsic affinity of factors for DNA.

To examine factor DNA binding in the context of developmentally programmed changes in chromatin accessibility, we analyzed *in vivo *occupancy data for two regulatory factors: hunchback (HB) at stage 9, at which time this factor is expressed in neuroblasts [71], and Medea (MED) at stages 10 and 14, which is expressed in all cells during embryogenesis, but is activated only in changing subsets of cells in response to transforming growth factor-β signaling [72-74].

**Figure 6:**
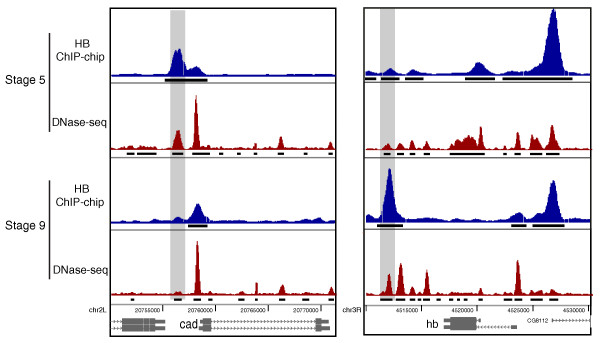
**Levels of HB factor occupancy and DNaseI accessibility change between developmental stages**. The level of hunchback (HB) binding and DNaseI accessibility to the *Caudal *(*cad*; left) and *hb*; right) genes are shown at stages 5 and 9. The figure is labeled using the same conventions in Figure 1 except that the locations of the regions above the ChIP-chip 1% FDR threshold are indicated by black horizontal lines beneath the continuous traces of ChIP-chip scores. Additional file 9 shows similar results for Medea (MED).

Both MED and HB exhibit temporal changes in occupancy, which visualization at individual gene loci suggests accompany programmed changes in chromatin accessibility (Figure 6; Additional file 9). A larger scale quantification of the change in factor binding shows that, between stage 5 and stages 9, 10 or 14, the correlation between binding levels for a given factor genome-wide range between r = 0.33 and r = 0.83, whereas the correlation between biological replicates at the same stage is r = 0.93 (Additional file 10). At most regions, therefore, the changes in levels of binding between stages for a protein are moderate, but are clearly distinguished from experimental variability between biological replicates.

**Figure 7:**
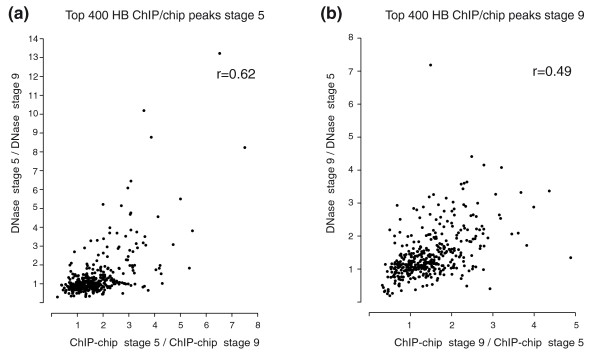
**Temporal changes in levels of HB occupancy correlate with changes in DNaseI accessibility**. We identified the 1-kb regions ±500 bp of the peak nucleotide of binding for each of the 400 regions most highly bound by HB at **(a) **stage 5 and **(b) **stage 9. (a) Scatter plot of the ratio of ChIP-chip scores at stage 5 over those at stage 9 (x-axis) versus the ratio of DNase-seq scores at stage 5 over those at stage 9 (y-axis). (b) Scatter plot of the ratio of ChIP-chip scores at stage 9 over those at stage 5 (x-axis) versus the ratio of DNase-seq scores at stage 9 over those at stage 5 (y-axis). The Pearson correlation coefficients (*r*) for each comparison are shown in the top right of each panel. See Additional file 11 for similar plots for MED.

To quantify the relationship between these temporal changes in factor occupancy and alterations in genome accessibility, we focused on the 400 most highly bound genomic regions at each stage. We then calculated for each highly bound region the ratio of ChIP-chip scores between pairs of stages for a factor and separately the ratio of the density of DNaseI cleavage between the same stages and then took the correlation between these two ratios (Figure 7). An advantage of this analysis strategy is that taking ratios within each data class first will greatly reduce any systematic bias introduced by either experimental protocol. Thus, analyzing the ratios will allow a more accurate comparison between two data types. Representative results for HB are shown in Figure 7, which reveals a clear correlation between temporal changes in binding and temporal changes in accessibility. Significant correlations (r = 0.49 to 0.8, *P*-values all <0.001) were likewise observed for all six pairwise comparisons between factors and stages (Figure 7; Additional file 11). Although strong, these correlations should be regarded as minimum estimates of the degree to which accessibility influences binding as remaining experimental biases in the data not removed by taking ratios will prevent a complete correlation.

## Discussion

We have shown that the phenomenon of widespread, overlapping patterns of DNA binding by different sequence-specific transcription factors in *Drosophila *embryos is tightly linked in a quantitative manner to DNA accessibility in chromatin. First, averaged across the entire euchromatic genome, the level of DNA binding *in vivo *at recognition sequences with similar intrinsic affinity for a given factor is much higher in accessible versus inaccessible chromatin for all 16 factors for which all corresponding data are available (Figure 4). Within highly accessible regions, the thousands of higher affinity recognition sequences for a single factor are generally the most highly occupied *in vivo*, but even the hundreds of thousands of moderate affinity sites are generally bound at higher levels than similar sites in less accessible regions. Second, the degree of chromatin accessibility is much more highly correlated with *in vivo *occupancy than with occupancy predicted from *in vitro *affinity measurements using purified protein and naked DNA (Figure 5). Third, there is a high quantitative correlation between programmed changes in accessibility during embryogenesis and changes in the level of factor DNA binding (Figure 7). Since the accessibility experienced by transcription factors must approximate that experienced by DNaseI, the high correlation between the experimentally measured alterations in factor DNA binding and DNaseI digestion suggests that altered chromatin accessibility is the dominant determinant of the change in binding, as opposed to other potential influences such as direct heteromeric cooperative interactions.

All of these results support a previously proposed 'widespread binding' model, which was initially based on comparisons between *in vivo *UV crosslinking data for different classes of homeoproteins and *in vitro *DNA binding, genetic, restriction enzyme accessibility, and target gene expression data [2,3,14,41,46]. In this model, regulatory factors are expressed at sufficiently high concentrations in cells that they can detectably occupy their recognition sequences in highly accessible chromatin without the aid of physical cooperative interactions with other proteins. Given the broad DNA recognition properties of animal transcription factors [23], this would inevitably lead to highly overlapping, graded binding of unrelated factors, with the lowest levels of binding being non-functional [2,3,14,41,46].

Computational modeling conducted in parallel to the studies presented here lends further credence to this model [75]. Using a generalized hidden Markov model, quite accurate quantitative predictions of the patterns of ChIP-seq *in vivo *DNA binding for five of the early *Drosophila *regulators can be made using only *in vitro *DNA binding and DNaseI accessibility data as input. No potential heteromeric interactions could be found in the model that would improve the prediction of DNA binding by these proteins, which are known to function in concert on a common pool of CRMs. Analysis of chromatin accessibility before and after induction of DNA binding of glucocorticoid receptor (GR) in different cell types also supports the widespread binding model. Not withstanding the fact that up to 12 to 15% of the regions bound by this pioneering transcription factor are inaccessible prior to induction, the remaining GR recognition sites in the genome that become bound are accessible prior to induction, with the different locations of GR binding between cell types largely correlating with the altered locations of accessible DNA [76].

The widespread binding model incorporates long-standing predictions that, given the relatively high concentrations of transcription factors and DNA in cells, the majority of factor molecules not bound at high levels to functional targets should be bound instead at lower densities to any accessible parts of the genome [77,78]. These thermodynamic arguments are supported by various lines of evidence suggesting that the concentration of free, unbound factor molecules in nuclei is indeed much lower than suggested by the number of molecules present [79-82]. Such predictions were originally made for the Lac repressor in *Escherichia coli *and assumed that genome-wide, low occupancy binding would result from the sequence-independent, electrostatic affinity of transcription factors for DNA (K_D _approximately 10^-6 ^M). Given the broad sequence-specific recognition properties of most animal transcription factors, however, it is likely that most accessible genomic regions will contain moderate or high affinity (K_D _< 10^-8 ^M) recognition sites for many of these proteins [23,83]. The factors whose *in vivo *binding we have examined are typically expressed at tens of thousands of molecules per cell [1,84] (BDTNP, unpublished data). Thus, thermodynamically, most of these molecules are likely to significantly occupy accessible moderate or high affinity recognition sequences, rather than being bound via an electrostatic, sequence-independent interaction. Indeed, even genomic regions bound at low levels *in vivo *are enriched for specific recognition sequences of a range of affinities ([3,14] and this paper).

DNA recognition sites for factors that would interfere with the proper regulation of a nearby gene will be actively selected against [85]. Low level binding at fortuitously occurring sites that does not lead to biologically significant transcriptional effects, in contrast, would not be subject to negative selection, and is consistent with the high amount of apparently incidental binding of factors detected *in vivo *[1-3,14,17].

Our analysis does not rule out an important role for direct heteromeric cooperative interactions between transcription factors quantitatively modifying binding of these proteins at a subset of recognition sequences. Our results, however, set limits on the extent to which direct positive heteromeric cooperative interactions are likely to determine the overall distribution of factor binding in cells. Because accessibility must affect binding, the high quantitative correlation we have measured between accessibility and *in vivo *binding leaves only a modest role for direct cooperative interactions to further modify binding.

A much larger role for direct heteromeric interactions in targeting transcription factor binding has been invoked where it is assumed that the concentrations at which factors are expressed in cells are too low to allow significant occupation of functional target sites without such interactions [26-30]. This 'direct cooperativity model' is associated with the idea that factors each bind and regulate a limited number of largely different genes, even in the same cell type (for example, [29]), and that even factors with similar intrinsic DNA recognition properties are targeted to different genes (for example, [26,30]). Based on the evidence presented here and the growing recognition that transcription factors bind a wide array of genomic regions in many animals and cell types [1-19], the direct cooperativity model may apply to a relatively limited set of factors and circumstances.

The occurrence of statistically significant local clusters of recognition sites for multiple transcription factors in a subset of CRMs modules (for example, [86-92]) could be taken as evidence for the direct cooperativity model. Such preferential clustering, however, could also result because of post-DNA-binding synergistic cooperativity between factors that does not significantly influence their targeting to DNA but instead influences members of the general transcriptional machinery [46,86,93]. Thus, the arrangement of recognition sites in the genome, while highly informative in detecting putative regulatory elements, cannot itself distinguish between different factor targeting mechanisms.

In addition to the long-standing evidence that nucleosomes inhibit the binding of transcription factors at some DNA regions *vivo *(reviewed by [32,40]), genome-wide studies have increasingly shown an association between regions bound by factors *in vivo *and features of chromatin structure, such as histone modifications, nucleosome content or accessibility [12,25,42-45,94-101]. These studies, however, have not shown that functionally distinct factors show a quantitative continuum of function and binding at common regions; nor observed a high quantitative correlation between DNA accessibility and factor binding; nor considered the classic thermodynamic predictions of Lin and Riggs [77] and Peter von Hippel [78]; nor sought to distinguish between the 'widespread binding' and the 'direct cooperativity' models for transcription factor targeting. Most of these studies have generally looked at the association qualitatively. In addition, the studies in yeast have not measured accessibility directly, but have attempted to infer it from ChIP-chip studies of nucleosome occupancy or nucleosome position sequence data [42], which will likely lead to some inaccuracy as genome accessibility is the product of all proteins bound to DNA and also high order chromatin structures. Our results thus highlight the importance of both measuring and considering the quantitative nature of factor binding and genome accessibility and of attempting to distinguish between alternative targeting models.

Finally, while our analysis does not address how the distribution of accessible regions in the genome is itself established, it is consistent with the indirect cooperativity model proposed by others in which different transcription factors mutually aid each other's binding to DNA by displacing a nucleosome without physically interacting with each other [47-56]. Indirect cooperativity, we suggest, implies that factors are expressed at a sufficiently high concentration in cells that they can occupy their recognition sites without the aid of direct protein-protein interactions with other proteins. It also predicts a high overlap in the genomic regions bound by transcription factors once the broad intrinsic DNA recognition properties of these proteins are taken into account. Most factors would be expected to contribute only a small part to determining the overall pattern of chromatin accessibility in this model, whereas chromatin accessibility would be expected to play a large role in determining the pattern of binding of each factor, when each is considered individually. The emerging picture is of a dynamic interplay between nucleosomes and sequence-specific DNA binding proteins (along with the remodeling/modification enzymes that they recruit) that mutually determine each other's binding patterns [34,35,37-40,57,58].

## Conclusions

Using the *Drosophila *embryo as a model system, we have provided a uniquely detailed, quantitative comparison between DNA accessibility and regulatory transcription factor occupancy *in vivo*. These analyses support a long-standing 'widespread binding' model [14,41,46,77-79,102], which suggests that animal regulatory factors are generally expressed at sufficiently high concentrations in cells that they can detectably occupy their recognition sequences in highly accessible chromatin without the aid of physical cooperative interactions with other proteins. Given the broad DNA recognition properties of animal transcription factors [23], this should inevitably lead to highly overlapping, graded binding of unrelated factors, with the lowest levels of binding being non-functional, consistent with extensive *in vivo *DNA binding and regulatory data in *Drosophila *[1-3,14,17,19,46]. This simple thermodynamic model predicts that similar widespread, overlapping DNA binding by many different regulatory transcription factors will be found in all animal cells.

## Materials and methods

### ChIP-chip of HB and MED in late stage embryos

Embryos were collected in population cages for 1 hour, and then allowed to develop to the required stage before being harvested and fixed with formaldehyde [14,65]. Chromatin was purified and ChIP-chip experiments were performed using affinity purified antibodies against HB and MED as described previously [14,17]. The data were processed as before to determine 1% FDR and 25% FDR bound regions and peaks using the symmetric null test [14] (Figure 2). All raw microarray data (CEL files) have been deposited at ArrayExpress [ArrayExpress: E-TABM-1021], and details of the locations of the 1% and 25% FDR bound regions are provided as Additional file 12. In addition, these and more processed forms of the data are available from the BDTNP's public web site [103].

### Determining the intersection of 5% FDR accessible regions and peaks

The raw DNase-seq DNA sequence tag data are from Thomas *et al*. ('Dynamic reprogramming of chromatin accessibility during *Drosophila *embryo development', submitted), which used methods described in [41,62,104] to generate the data. For convenience, the NCBI Sequence Read Archive accession numbers for these data are also provided here: [NCBI SRA: STUDY SRP002474, NCBI SRA EXPERIMENTS SRX020691 to SRX020700] for stage 5 rep 1 to stage 14 rep 2, respectively). As described (S Thomas *et al*., submitted), DNaseI accessible regions were defined using a scan statistic that identified regions with DNaseI cleavage densities that were significantly above the local 50 kb background. Regions at 5% FDR were identified (Additional files 2 and 3). Peaks in accessibility were identified from local maxima in tag density within 75 bp of a given 20-bp sliding window across each accessible region (Additional files 2 and 3). The conservatively defined set of accessible regions and peaks in accessibility that were found in both replicates at each stage were used for subsequent analysis (for example, Additional files 5, 6 and 7).

### Correlating factor binding and genome accessibility

The locations of 1% FDR ChIP-chip peaks for 21 factors at stage 5 were obtained from previously published data [14,17,103] [Array Express; E-TABM-736]. The percentage of ChIP-chip peaks overlapped by accessible chromatin for each factor at stage 5 (Additional file 5) was calculated by adding the number of instances either where the 1-kb ChIP-chip peak was overlapped by an accessible region by at least 200 bp or where a ChIP-chip peak entirely encompassed a 5% FDR DNaseI accessible region, and dividing by the total number of 1% FDR ChIP-chip peaks. The significance of this coverage was assessed using two separate methods, a simple hypergeometric model and the Genome Structure Correction (GSC) statistic [105]. The hypergeometric model assessed the likelihood of set A to include 'q' base pairs of overlap with set 'B', assuming n draws without replacement from the genome where n is the base-pair coverage of set A. GSC is a more complex bootstrapping method specifically designed to calculate probabilities of overlap for sets of genomics features. For both tests it was impossible to determine with any further accuracy the probabilities of overlaps for each factor with greater significance than the *de minimus *probability of 1 × 10^-16^.

To determine what fraction of the accessible regions was covered by one or more factors (Additional file 5), all of the single-nucleotide locations of 1% FDR ChIP-chip peaks [14,17] for all factors were merged and padded on either end by 500 bp to account for imprecision in the location of each peak. Peaks in DNaseI accessibility in stage 5 embryos were ranked from largest to smallest and divided into cohorts of 1,000 peaks. If any of the merged ChIP regions fell within 75 bp of a peak in accessibility, then that DNaseI peak was said to be 'covered' by a ChIP factor. The fraction of peaks that were bound by any of the factors was calculated as the number of 'covered' peaks divided by the number of peaks per cohort.

The 25% FDR ChIP-chip peaks for each factor were ranked from largest to smallest and divided into cohorts of 200 peaks (Figure 3; Additional file 7). The maximum DNaseI density for stage 5 and 14 embryos within 500 bp of each ChIP-chip peak was recorded as was whether or not that peak overlapped a stage 5 DNaseI accessible region. The number of ChIP-chip peaks in each cohort that overlapped a stage 5 accessible region divided by the number of peaks in each cohort was calculated to determine the percent of ChIP-chip peaks in each cohort that were in accessible regions. The median and 95% confidence intervals of maximum DNaseI densities for the ChIP-chip peak cohorts were calculated with R's box plot function [106].

### Measuring the effect of accessibility and intrinsic factor specificity on *in vivo *occupancy

PWMs for 16 transcription factors have previously been collated [17] from various *in vitro *SELEX and DNaseI footprinting experiments that used purified transcription factor protein and naked DNA [70] (BDTNP, unpublished data). For convenience these are provided in Additional file 13. These PWMs were used to identify all DNA sequences that match them genome-wide at *P*-values <0.04 using Fimo [107]. For each factor, these recognition site occurrences were then divided into two groups depending on whether the matches were located within 5% FDR DNaseI accessible regions or whether they were in inaccessible chromatin. The recognition sites were then further broken down into cohorts in R based on *P*-values as follows:

For each cohort, the maximum ChIP-chip signal from the relevant factor within 250 bp of each sequence match was determined using input DNA normalized ChIP-chip scores calculated as Array hybridization signal for factor immunoprecipitation/Array hybridization signal for input crosslinked DNA (see Figure 2 in [14]) except that natural numbers, not log2, were used here. The 95% confidence interval about the median of these scores was calculated using R's box plot function (Figure 4; Additional file 8).

In addition, ten permutations of each original PWM were generated by shuffling the order of positions in the weight matrices for each permutation. If any permutation that matched any other of the randomly generated permutations for that factor or the normal PWM of one of the other 15 factors (*P *< 0.05 defined using Tomtom [108]) it was discarded and a new permutation was generated. The set of sequence matches to these scrambled PWMs were then identified throughout the genome, separated into those in open or closed chromatin and binned into groups based on affinity in the same manner as for the genuine motifs. The maximum ChIP-chip scores within 250 bp of each scrambled recognition site occurrence was determined and the median of this peak score was determined over the entire set of ten scrambled PWMs for each factor and the 95% confidence limits calculated as for the matches to the genuine PWMs (Figure 4; Additional file 8).

To correlate accessibility with ChIP-chip scores (Figure 5a), peaks in accessibility at stage 5 were annotated with maximum input DNA normalized ChIP-chip scores within 75 bp of each peak for the 16 factors with well-characterized *in vitro *binding specificities (Figure 4; Additional file 8). The peaks were ranked by accessibility and the correlation between level of accessibility and ChIP-chip score was calculated using R's Pearson correlation function. The DNaseI peaks were then ranked, separated into cohorts of 200 similarly accessible peaks and the median peak in ChIP-chip signal for each cohort was determined and plotted using R's heat map function scaling rows to account for inherent differences in ChIP-chip signal between factors. A similar process was used to correlate accessibility with the presence of recognition sites for each of the 16 factors (Figure 5b). The same PWMs for the factors derived from *in vitro *DNA binding data, described above, were employed to identify all sequence matches to these matrices within 75 bp of peaks of accessibility with *P *< 0.003 using Fimo [107] (that is, matches that fell into at least the -3 cohort from Figure 4). The correlation between the level of accessibility and the number of PWM matches was calculated using R's Pearson correlation function. For each factor, the peaks in accessibility were ranked and divided into cohorts of 200 and the sum of all recognition sites was added over each cohort and plotted in R using the heat map function, while scaling rows to one another in order to account for differences in information content between PWMs.

### Correlating temporal changes in factor occupancy and DNA accessibility

Scatter plots and Pearson correlations were generated using R (Figure 7; Additional files 10 and 11). Peaks in ChIP-chip data for HB2 antibody above the 25% FDR threshold were annotated by the maximum ChIP-chip signal for HB 1 and HB 2 within 500 bp of each peak [17], and these two replicate input DNA normalized ChIP-chip scores were plotted against each other and a correlation coefficient calculated (Additional file 10). This same process was used to assess the correlation between maximum HB 2 ChIP-chip signal from stage 5 embryos compared to HB 2 ChIP-chip signal from stage 9 embryos, as well as to compare MED ChIP-chip signals from stage 5, 10 and 11 embryos. This process was also used to determine if the changes in ChIP-chip signal were correlated with changes in chromatin accessibility at the same genomic regions (Figure 7; Additional file 11). For these plots, the ratio between input DNA normalized ChIP-chip scores for stage X and scores for stage Y was plotted against the ratio between DNAse-seq density for stage X and density for stage Y for the following six pairwise comparisons: HB 2 stage 5/HB 2 stage 9; HB 2 stage 9/HB 2 stage 5; MED stage 5/MED stage 10; MED stage 5/MED stage 14; MED stage 10/MED stage 5; and MED stage 14/MED stage 5.

## Abbreviations

BDTNP: Berkeley *Drosophila *Transcription Network Project; bp: base pair; cad: caudal; ChIP-chip: chromatin immunoprecipitation followed by microarray analysis; CRM: *cis*-regulatory module; D: Dichaete; DNase-seq: DNaseI digestion of nuclei followed by high throughput DNA sequencing; eve: even-skipped; FDR: false discovery rate; GR: glucocorticoid receptor; HB: hunchback; MED, Medea; PWM: position weight matrix; TWI: Twist.

## Authors' contributions

XL, ST, MBE, JAS and MDB conceived and designed the experiments and analyses and wrote the paper. XL and PJS performed the wet laboratory experiments. XL, ST, JAS and MDB analyzed the data. All authors read and approved the final manuscript.

## Supplementary Material

Additional file 1 **Replica DNase-seq data closely agree**. Click here for file

Additional file 2 **Summary of 5% FDR accessible regions in euchromatic DNA for stage 5, 9, 10, 11 and 14 embryos**. Click here for file

Additional file 3 **5% FDR accessible regions in the euchromatic genome for stage 5, 9, 10, 11 and 14 embryos**. Click here for file

Additional file 4 **ChIP-chip input crosslinked DNA is not appreciably enriched in either highly bound or highly accessible genomic regions**. Click here for file

Additional file 5 **The overlap between 1% FDR ChIP-chip peaks versus 5% FDR accessible regions**. Click here for file

Additional file 6 **Most highly accessible regions are bound by regulatory factors**. Click here for file

Additional file 7 **The level of transcription factor occupancy correlates with the degree of DNaseI accessibility**. Click here for file

Additional file 8 **Comparison of ChIP-chip scores for occurrences of DNA recognition sequences in accessible versus closed chromatin regions**. Click here for file

Additional file 9 **Levels of MED factor occupancy and DNaseI accessibility change between developmental stages**. Click here for file

Additional file 10 **Change in DNA binding levels *in vivo *between developmental stages**. Click here for file

Additional file 11 **Temporal changes in levels of MED occupancy correlate with changes in DNaseI accessibility**. Click here for file

Additional file 12 **1% and 25% FDR ChIP-chip bound regions for HB at stage 9 and MED at stages 10 and 14**. Click here for file

Additional file 13 **Position weight matrices of factors' intrinsic DNA recognition properties used**. Click here for file
