# Supplementary material for: The role of chromatin accessibility in directing the widespread, overlapping patterns of Drosophila transcription factor binding
Source: Genome Biol. 2011 Apr 7;12(4):R34. doi: 10.1186/gb-2011-12-4-r34 (PMC3218860; doi:10.1186/gb-2011-12-4-r34)

Additional data file 1. Replica DNase-seq data closely agree. DNaseI accessibility at stage 5 is shown for 75 bp windows of sequence tag density (red) for two independent biological replicas in a region of the genome spanning the eve gene. The locations of those accessible sequences that are above the 5% FDR threshold in both replicas are also shown (black bars). At the bottom, the locations of major RNA transcripts are indicated (grey). The results are consistent with earlier analyses of the same data (Thomas et al: "Dynamic Reprogramming of Chromatin Accessibility During Drosophila Embryo Development", submitted).

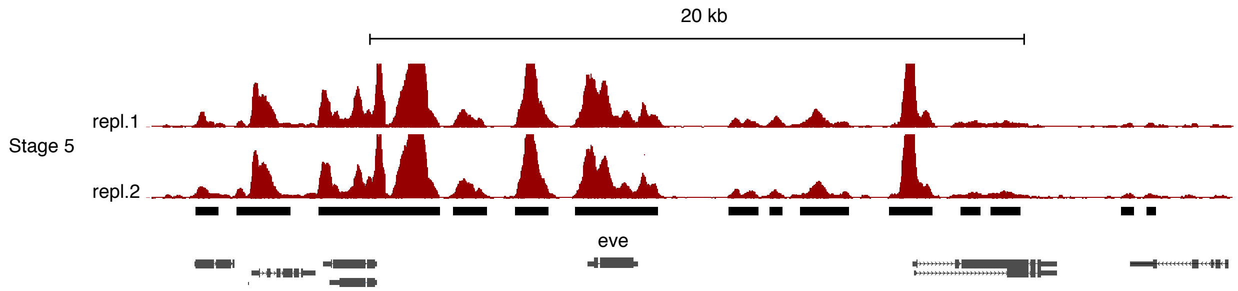

Supplement: Additional file 1 — Replica DNase-seq data closely agree. [file gb-2011-12-4-r34-S1.PDF]
