# Supplementary material for: The role of chromatin accessibility in directing the widespread, overlapping patterns of Drosophila transcription factor binding
Source: Genome Biol. 2011 Apr 7;12(4):R34. doi: 10.1186/gb-2011-12-4-r34 (PMC3218860; doi:10.1186/gb-2011-12-4-r34)
Supplement: Additional file 2 — Summary of 5% FDR accessible regions in euchromatic DNA for stage 5, 9, 10, 11 and 14 embryos. [file gb-2011-12-4-r34-S2.PDF]

Additional data file 2. Summary of 5% FDR DNaseI accessible regions in euchromatic DNA for stage 5, 9, 10, 11 and 14 embryos.

Embryo stage	Replica or intersection	Number of 5% FDR DNaseI accessible regions	Genome covered by accessible regions	Number of peaks in accessible regions	Genome covered by peaks	Correlation between replicas
5	1	26,671	18.4 Mb	27,787	4.2 Mb	0.98
	2	24,270	17.3 Mb	25,503	4.0 Mb	
	intersection	22,694	14.5 Mb	18,940	2.3 Mb	
9	1	20,097	14.2 Mb	21,497	3.2 Mb	0.97
	2	18,331	13.7 Mb	26,074	3.9 Mb	
	intersection	16,217	13.2 Mb	21,513	3.2 Mb	
10	1	27,403	15.9 Mb	29,343	4.4 Mb	0.95
	2	30,019	17.9 Mb	31,732	4.8 Mb	
	intersection	23,766	13.1 Mb	19,868	2.4 Mb	
11	1	22,018	15.5 Mb	23,239	3.5 Mb	0.98
	2	24,343	16.4 Mb	25,556	3.8 Mb	
	intersection	19,743	12.8 Mb	16,665	1.9 Mb	
14	1	35,470	14.9 Mb	35,872	5.4 Mb	0.91
	2	28,781	14.0 Mb	19,582	4.4 Mb	
	intersection	23,065	10.3 Mb	19,582	2.2 Mb	

Genome coordinates for the 5% FDR accessible regions and peaks found in both replicas for each stage (intersection) are given in Additional data file 3.
