# Supplementary material for: The role of chromatin accessibility in directing the widespread, overlapping patterns of Drosophila transcription factor binding
Source: Genome Biol. 2011 Apr 7;12(4):R34. doi: 10.1186/gb-2011-12-4-r34 (PMC3218860; doi:10.1186/gb-2011-12-4-r34)

Additional data file 4. ChIP-chip input crosslinked DNA is not appreciably enriched in either highly bound or highly accessible genomic regions. A. The 1,000 genomic regions most highly bound by the transcription factor KR were identified from the rank list of KR1 1% FDR ChIP-chip bound regions at stage 5 [17]. These 1,000 genomic sequences were centered using their local maximum ChIP-chip score. The median array hybridization scores for the 5 kb regions flanking these peaks were determined for a series of 200 bp windows for either the purified cross-linked input DNA sample (black line) or the DNA immunoprecipitated using anti KR antibody 1 (orange line). **B.** The 1,000 most accessible regions of the genome were identified from the rank list of 5% FDR DNase-seq peaks at stage 5 (Additional data file 3). These 1,000 genomic sequences were centered using the local maximum DNaseI-seq score. The median array hybridization scores for the 5 kb regions flanking these peaks were determined for a series of 200 bp windows for either the purified crosslinked input DNA sample (black line) or the DNA immunoprecipitated using anti KR antibody 1 (orange line). Note that the ChIP-chip scores used in the remainder of the paper are normalized by dividing the factor IP scores, shown in this example for KR (orange lines), by the Input DNA scores (black lines), as described [14].

The figure shows that there is no appreciable enrichment of input DNA signal in either the regions highly bound by KR or the most accessible portions of the genome. This result differs from that of a similar analysis by Auerbach et al [66], presumably because of the differences in the DNA extraction procedures used, which are discussed in the text.

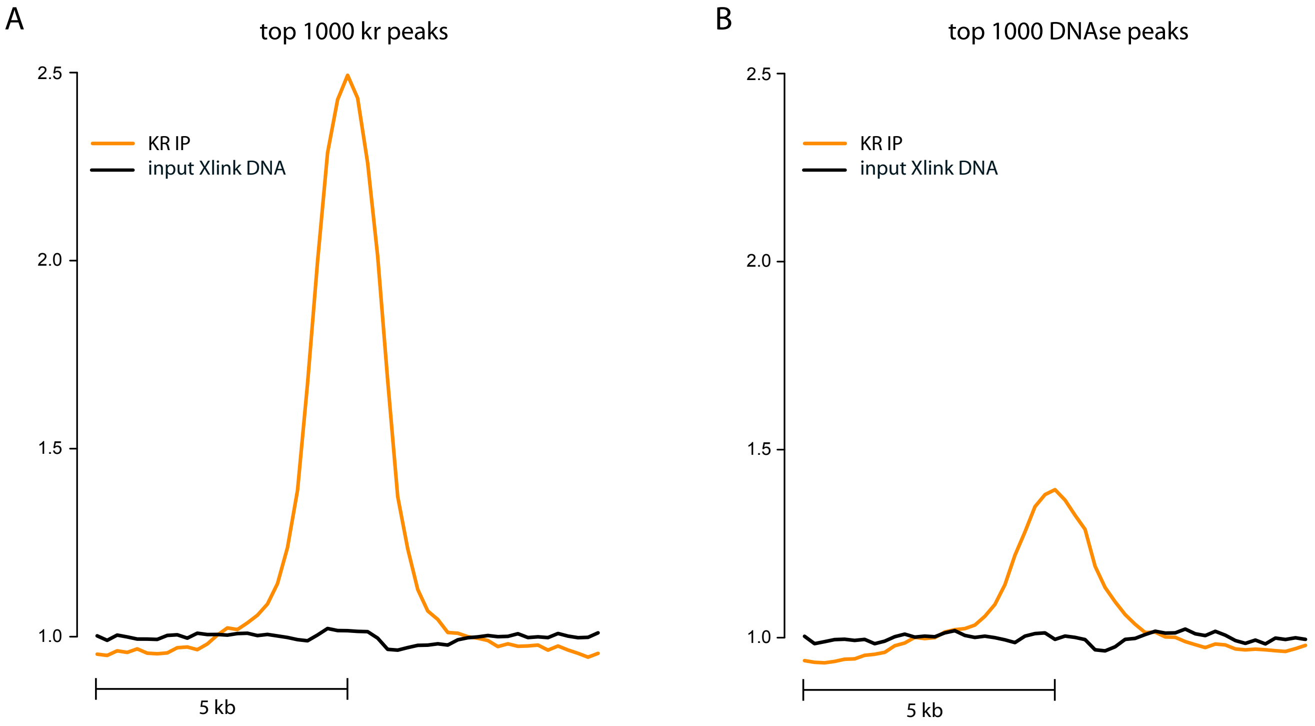

Supplement: Additional file 4 — ChIP-chip input crosslinked DNA is not appreciably enriched in either highly bound or highly accessible genomic regions. [file gb-2011-12-4-r34-S4.PDF]
