# Supplementary material for: The role of chromatin accessibility in directing the widespread, overlapping patterns of Drosophila transcription factor binding
Source: Genome Biol. 2011 Apr 7;12(4):R34. doi: 10.1186/gb-2011-12-4-r34 (PMC3218860; doi:10.1186/gb-2011-12-4-r34)
Supplement: Additional file 5 — The overlap between 1% FDR ChIP-chip peaks versus 5% FDR accessible regions. [file gb-2011-12-4-r34-S5.PDF]

Additional data file 5. The overlap between 1% FDR ChIP-chip peaks vs 5% FDR DNaseI accessible regions.

Regulatory class	Transcription factor antibody	Number of ChIP-chip peaks bound at 1% FDR [1]	% of ChIP-chip peaks overlapped by a 5% FDR accessible region [2]	probability of overlap (hypergeometric model)
A-P early	BCD 2	702	96	$<1 \times 10^{-16}$
	CAD 1	1,591	92	$<1 \times 10^{-16}$
	GT 2	1,070	94	$<1 \times 10^{-16}$
	HB 1	1,832	91	$<1 \times 10^{-16}$
	KNI 2	197	98	$<1 \times 10^{-16}$
	KR 2	4,084	72	$<1 \times 10^{-16}$
	HKB 1	1,012	94	$<1 \times 10^{-16}$
	TLL 1	429	97	$<1 \times 10^{-16}$
	D 1	6,452	87	$<1 \times 10^{-16}$
A-P pair rule	FTZ 3	403	83	$<1 \times 10^{-16}$
	HRY 2	2,729	89	$<1 \times 10^{-16}$
	PRD 1	2,061	83	$<1 \times 10^{-16}$
	RUN 1	921	93	$<1 \times 10^{-16}$
	SLP1 1	1,171	88	$<1 \times 10^{-16}$
D-V	DA 2	5,534	81	$<1 \times 10^{-16}$
	DL 3	9,358	76	$<1 \times 10^{-16}$
	MAD 2	204	74	$<1 \times 10^{-16}$
	MED 2	5,458	96	$<1 \times 10^{-16}$
	SHN 2	341	99	$<1 \times 10^{-16}$
	SHN 3	121	98	$<1 \times 10^{-16}$
	SNA 1	596	86	$<1 \times 10^{-16}$
	SNA 2	2,800	71	$<1 \times 10^{-16}$
	TWI 1	6,686	77	$<1 \times 10^{-16}$
	TWI 2	7,416	73	$<1 \times 10^{-16}$
General	Pol II H14	3,108	74	$<1 \times 10^{-16}$
	TFIIB	1,943	93	$<1 \times 10^{-16}$

[1] Number of ChIP-chip peaks taken from MacArthur et al, 2009 [17]

[2] 87% mean overlap for all factors
