# Supplementary material for: The role of chromatin accessibility in directing the widespread, overlapping patterns of Drosophila transcription factor binding
Source: Genome Biol. 2011 Apr 7;12(4):R34. doi: 10.1186/gb-2011-12-4-r34 (PMC3218860; doi:10.1186/gb-2011-12-4-r34)

Additional data file 6. Most highly accessible regions are bound by regulatory factors. The plot shows the percent of DNaseI peaks close to sequences bound by at least one sequence specific factor in non-overlapping cohorts of 1,000 peaks down the DNase-seq rank list. All DNaseI peaks within 575 bp of the peak nucleotide of binding of at least one factor were counted. The most accessible regions are to the left along the x-axis and results are plotted for all peaks of accessibility within 5% FDR DNaseI accessible regions. The ChIP-chip and DNase-seq data used are for stage 5 embryos. The locations of DNase-seq peaks are given in Additional data file 3.

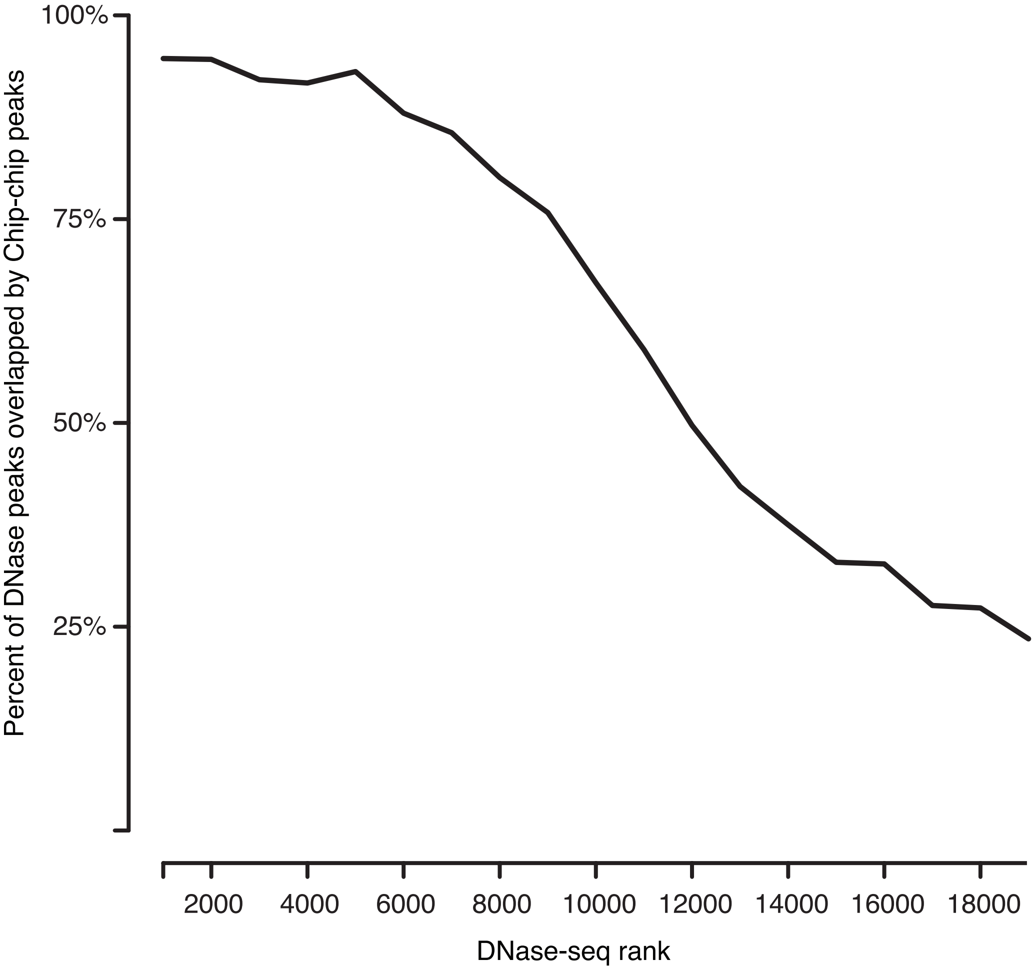

Supplement: Additional file 6 — Most highly accessible regions are bound by regulatory factors. [file gb-2011-12-4-r34-S6.PDF]
