# Supplementary material for: The role of chromatin accessibility in directing the widespread, overlapping patterns of Drosophila transcription factor binding
Source: Genome Biol. 2011 Apr 7;12(4):R34. doi: 10.1186/gb-2011-12-4-r34 (PMC3218860; doi:10.1186/gb-2011-12-4-r34)
Supplement: Additional file 7 — The level of transcription factor occupancy correlates with the degree of DNaseI accessibility. [file gb-2011-12-4-r34-S7.PDF]

Additional data file 7. The level of transcription factor occupancy correlates with the degree of DNaseI accessibility. The median DNase-seq tag density in non-overlapping cohorts of 200 ChIP-chip 1 kb peaks is shown down to the ChIP-chip rank list (continuous lines). The ChIP-chip data are from stage 5 embryos and the DNaseI accessibility data is from stages 5 (green) and 14 (purple). 95% confidence limit for the median DNaseI accessibility of each cohort is indicated. Shown also is the percent of ChIP-chip peaks that are overlapped by 5% FDR DNaseI accessible regions in stage 5 embryos (dashed green line). The regions most highly bound by transcription factors are to the left along the x-axis and results are plotted as far as the ChIP-chip 25% FDR cutoff. The location of the ChIP-chip 1% FDR threshold is indicated by a black, vertical dotted line. Results for all 21 regulatory transcription factors are shown.

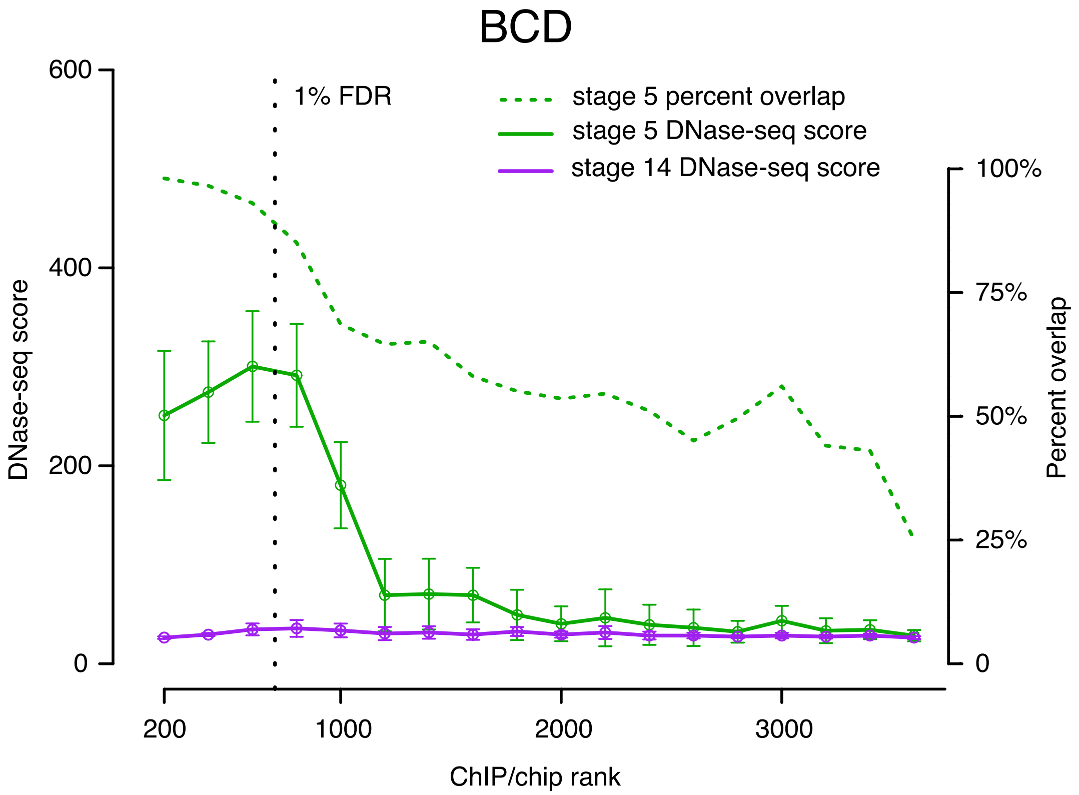

BCD
1% FDR
stage 5 percent overlap
stage 5 DNase-seq score
stage 14 DNase-seq score
DNase-seq score
600
400
200
0
Percent overlap
100%
75%
50%
25%
0
200
1000
2000
3000
ChIP/chip rank

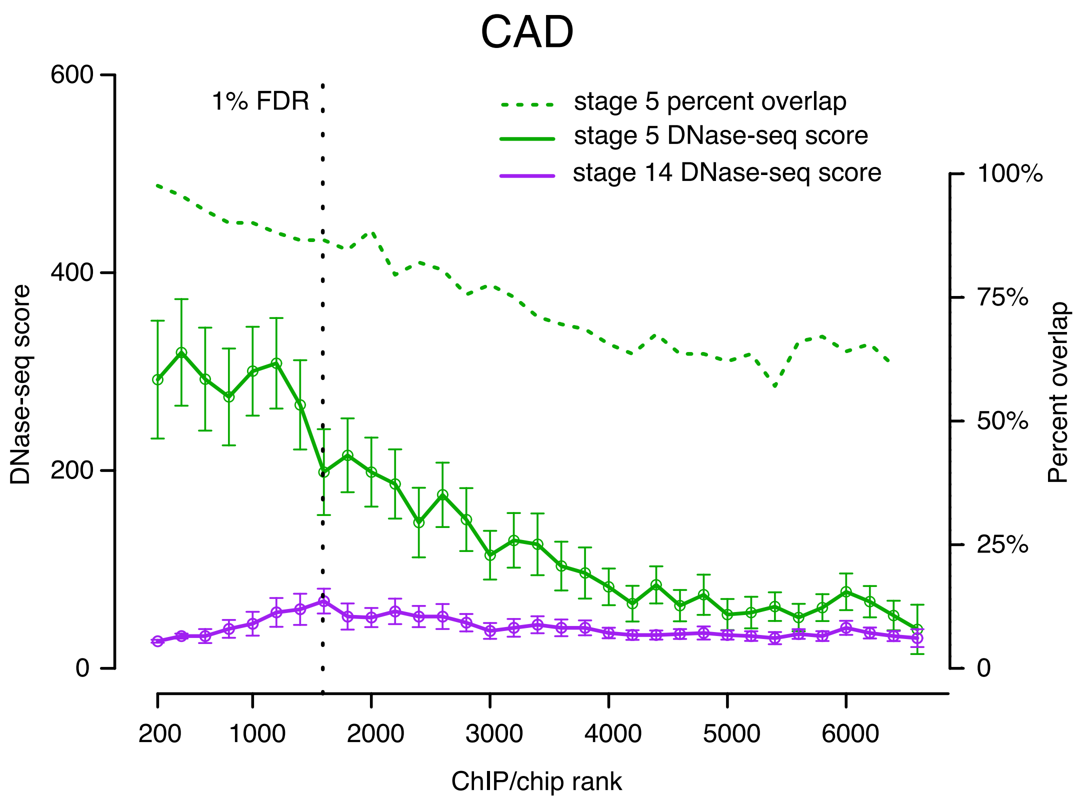

CAD
1% FDR
stage 5 percent overlap
stage 5 DNase-seq score
stage 14 DNase-seq score
DNase-seq score
Percent overlap
ChIP/chip rank
600
400
200
0
100%
75%
50%
25%
0
200
1000
2000
3000
4000
5000
6000

D

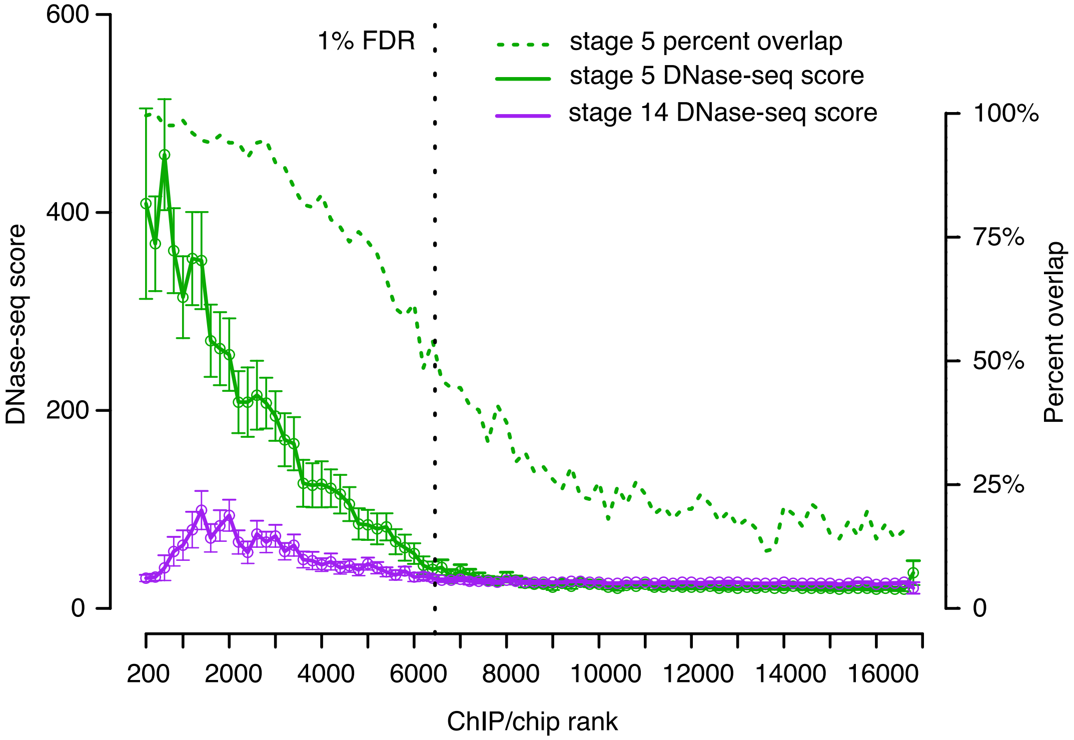

600
400
200
0
DNase-seq score
1% FDR
stage 5 percent overlap
stage 5 DNase-seq score
stage 14 DNase-seq score
100%
75%
50%
25%
0
Percent overlap
200
2000
4000
6000
8000
10000
12000
14000
16000
ChIP/chip rank

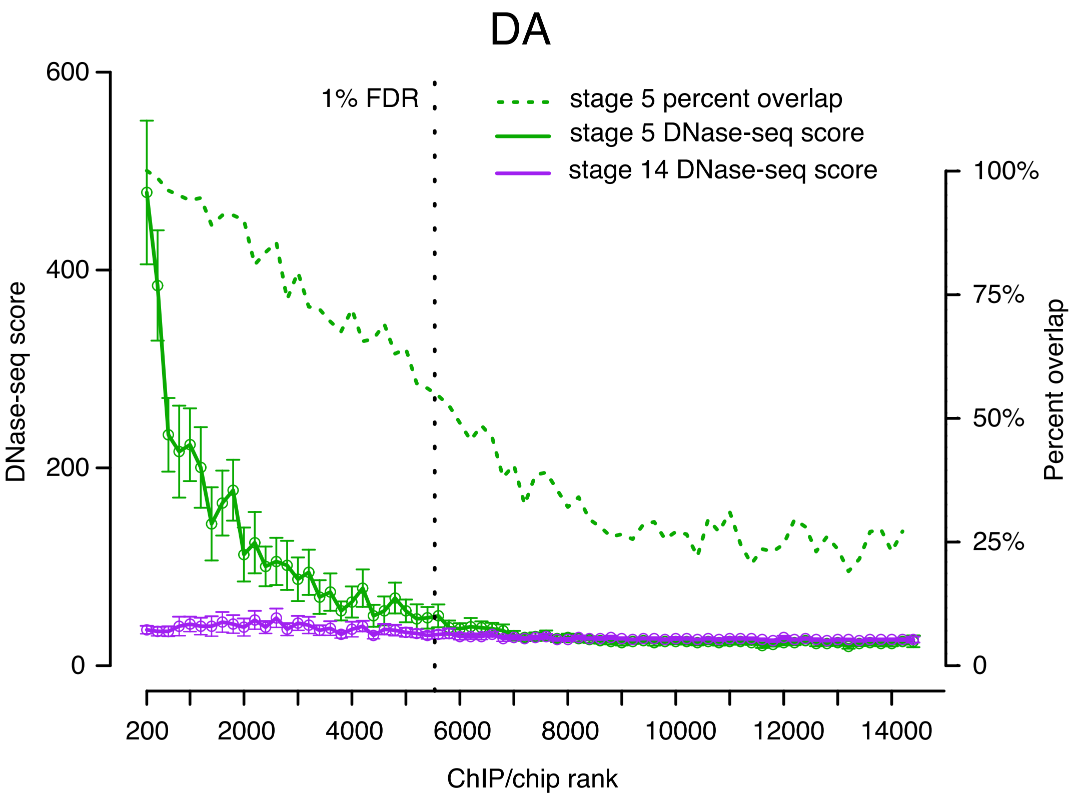

DA
600
400
200
0
DNase-seq score
1% FDR
stage 5 percent overlap
stage 5 DNase-seq score
stage 14 DNase-seq score
100%
75%
50%
25%
0
Percent overlap
200
2000
4000
6000
8000
10000
12000
14000
ChIP/chip rank

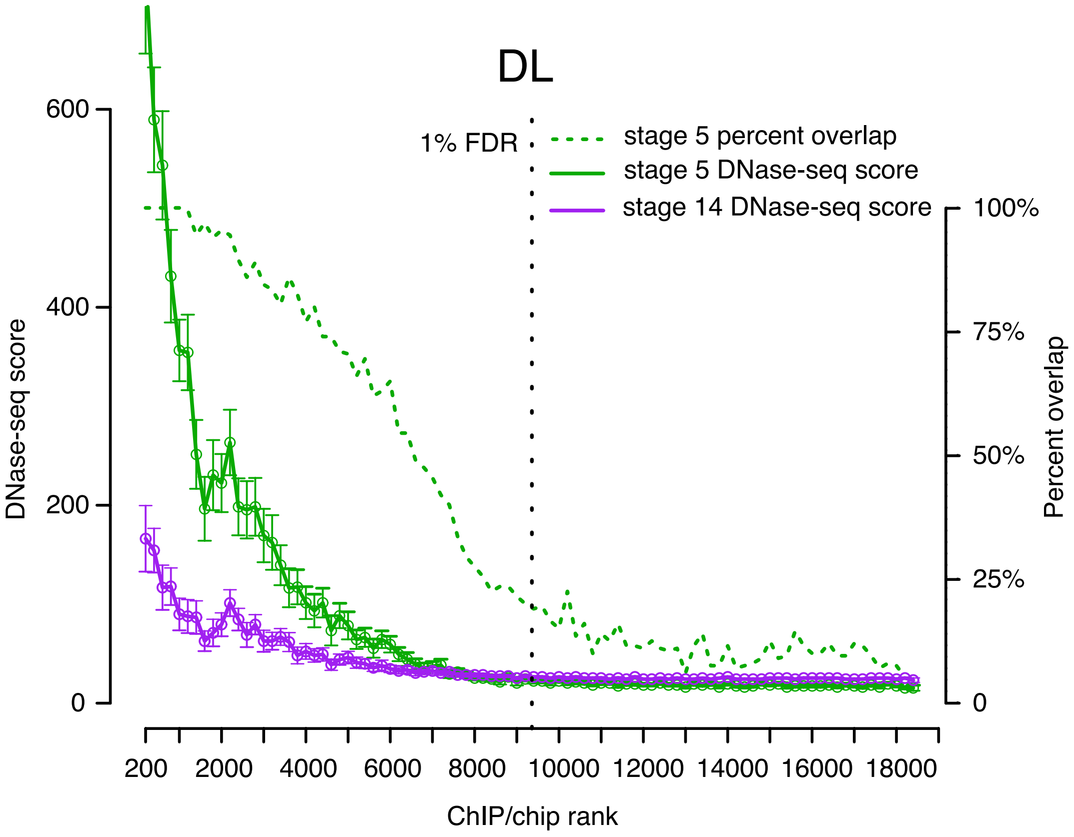

DL
1% FDR
stage 5 percent overlap
stage 5 DNase-seq score
stage 14 DNase-seq score
DNase-seq score
Percent overlap
ChIP/chip rank
0
200
400
600
0
25%
50%
75%
100%
200
2000
4000
6000
8000
10000
12000
14000
16000
18000

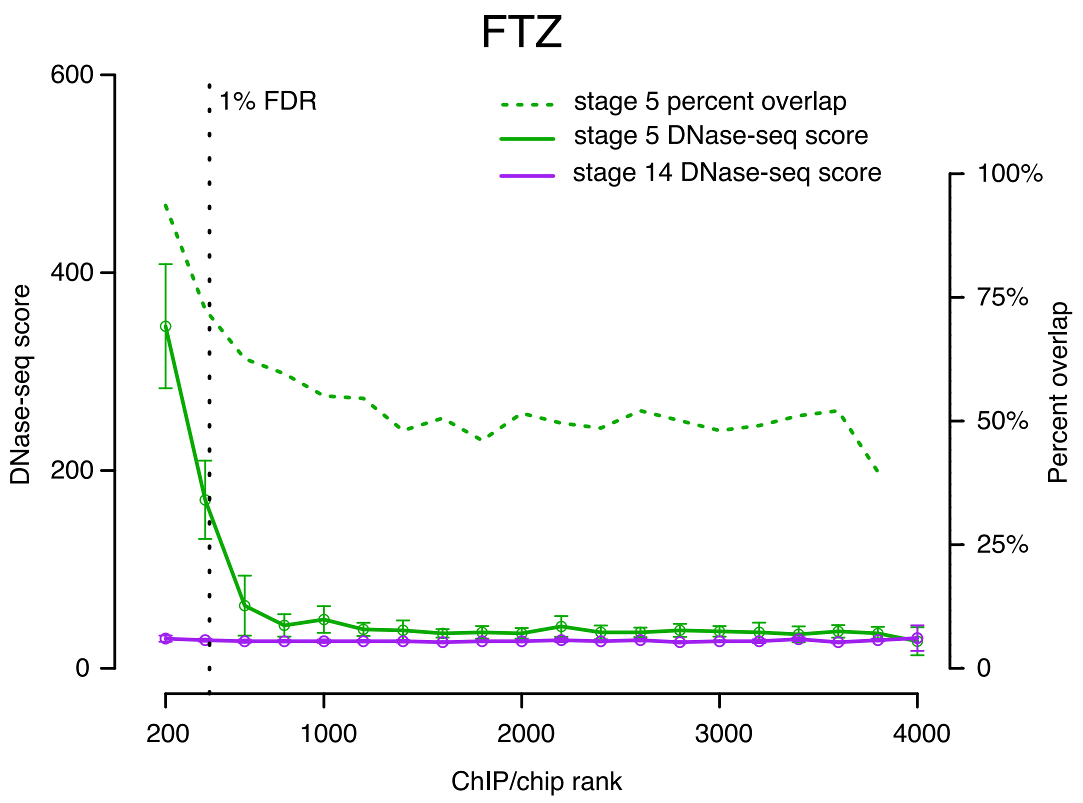

FTZ
1% FDR
stage 5 percent overlap
stage 5 DNase-seq score
stage 14 DNase-seq score
DNase-seq score
Percent overlap
ChIP/chip rank
600
400
200
0
100%
75%
50%
25%
0
200
1000
2000
3000
4000

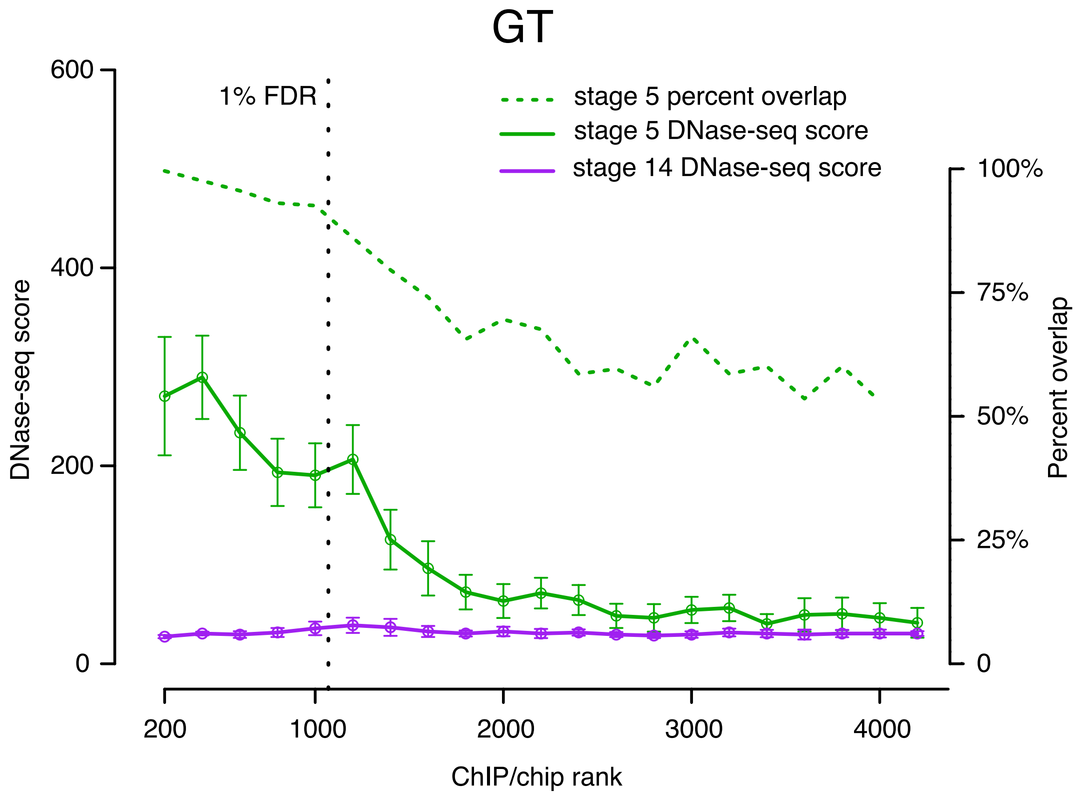
GT
1% FDR
stage 5 percent overlap
stage 5 DNase-seq score
stage 14 DNase-seq score
DNase-seq score
Percent overlap
ChIP/chip rank
600
400
200
0
100%
75%
50%
25%
0
200
1000
2000
3000
4000

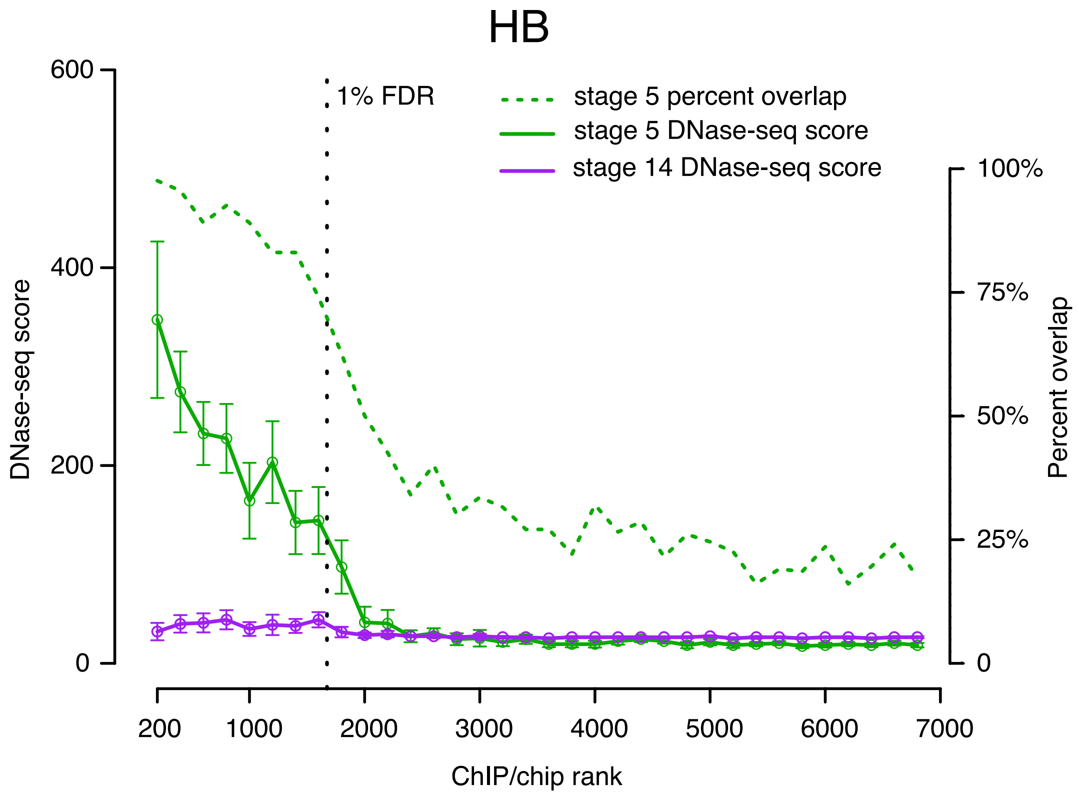
HB
600
400
200
0
DNase-seq score
1% FDR
stage 5 percent overlap
stage 5 DNase-seq score
stage 14 DNase-seq score
100%
75%
50%
25%
0
Percent overlap
200
1000
2000
3000
4000
5000
6000
7000
ChIP/chip rank

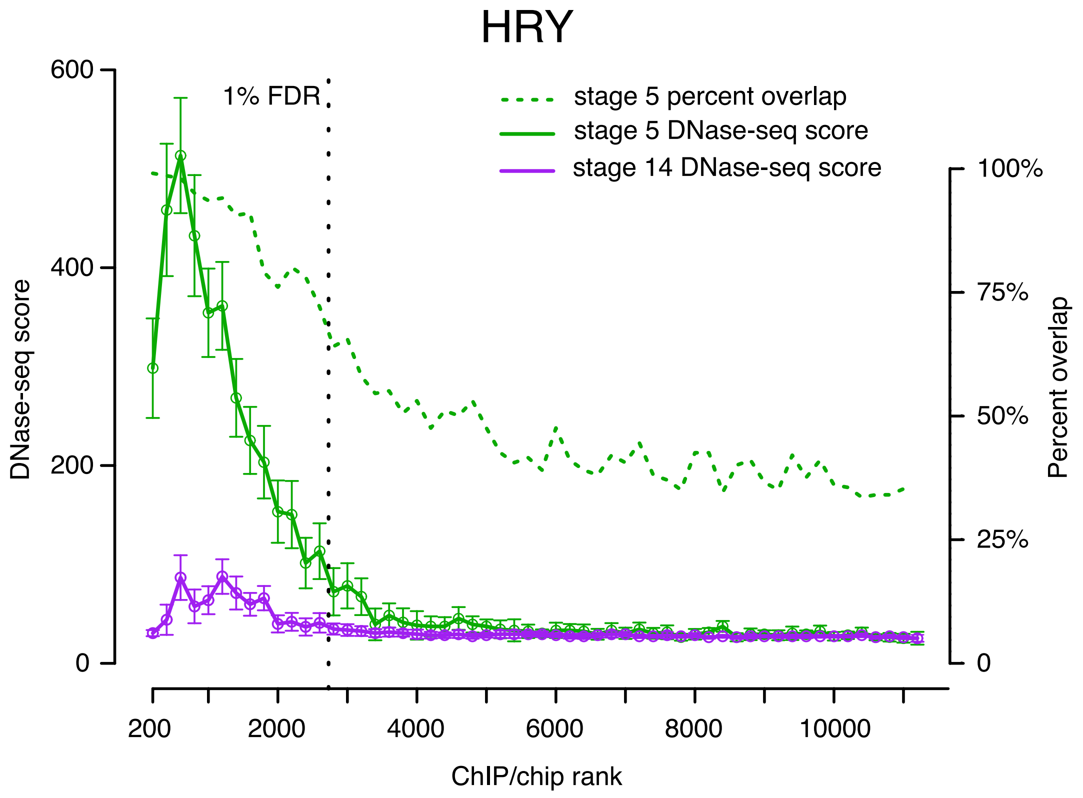

HRY
1% FDR
stage 5 percent overlap
stage 5 DNase-seq score
stage 14 DNase-seq score
DNase-seq score
Percent overlap
ChIP/chip rank
0
200
400
600
0
25%
50%
75%
100%
200
2000
4000
6000
8000
10000

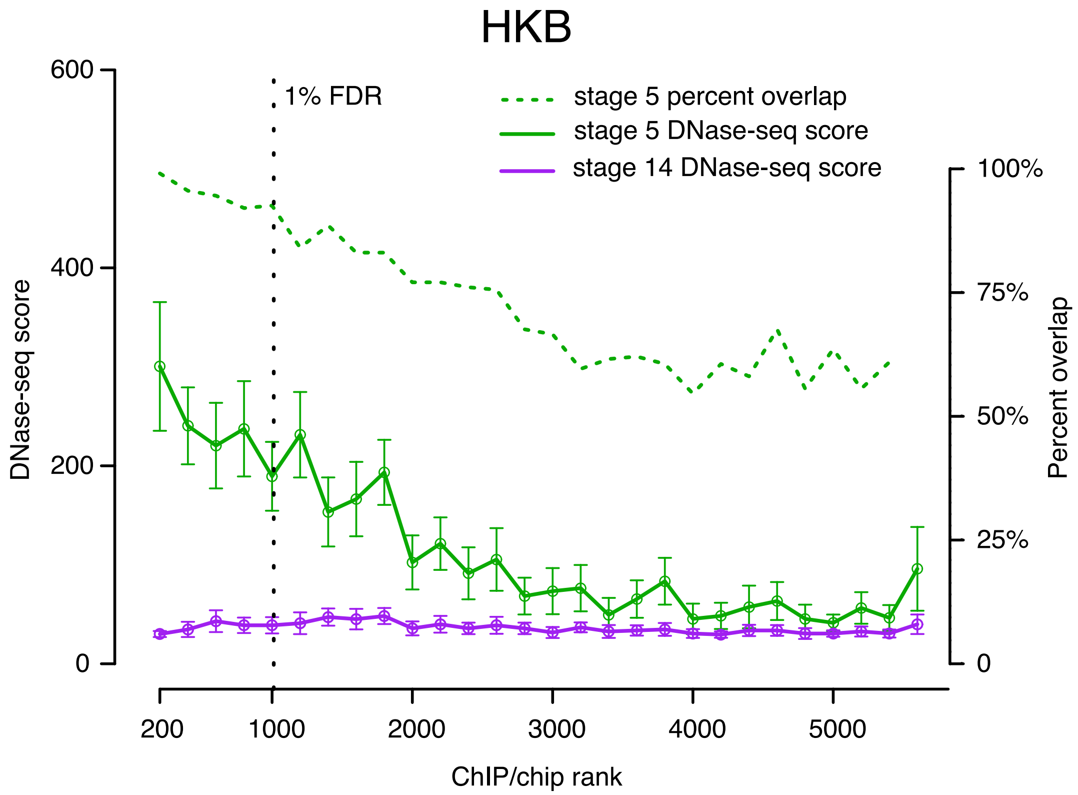

HKB
1% FDR
stage 5 percent overlap
stage 5 DNase-seq score
stage 14 DNase-seq score
DNase-seq score
600
400
200
0
Percent overlap
100%
75%
50%
25%
0
200
1000
2000
3000
4000
5000
ChIP/chip rank

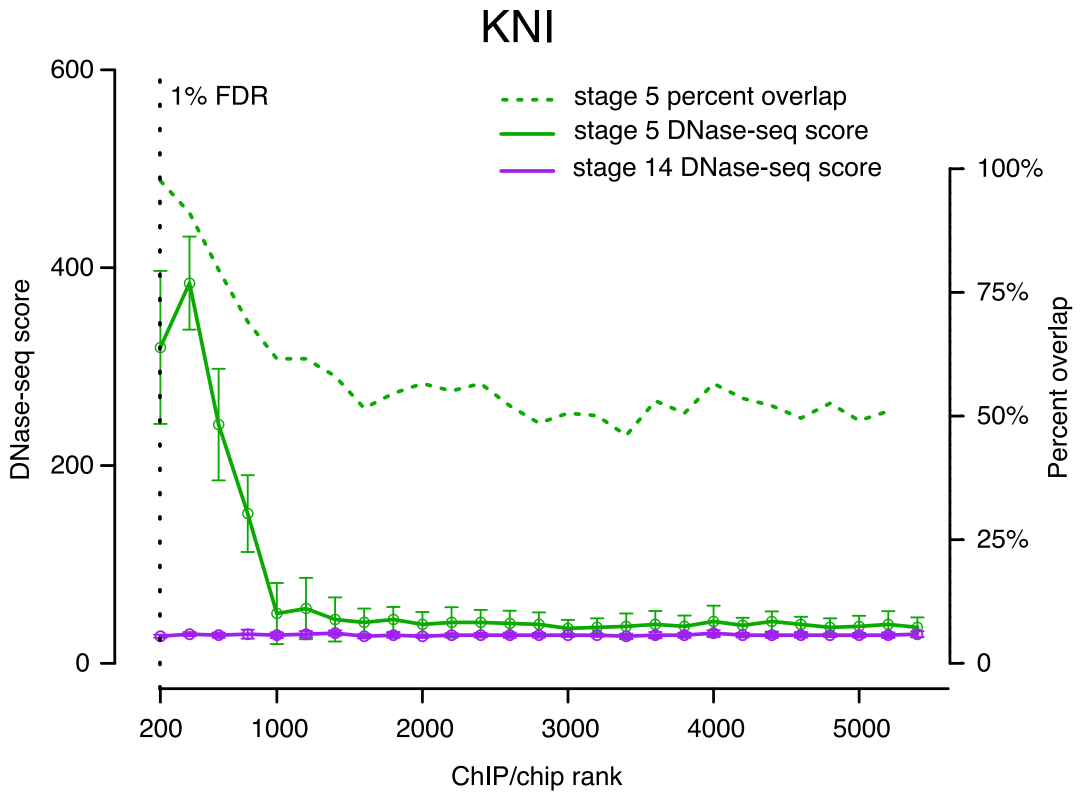
KNI
1% FDR
stage 5 percent overlap
stage 5 DNase-seq score
stage 14 DNase-seq score
DNase-seq score
Percent overlap
ChIP/chip rank
600
400
200
0
100%
75%
50%
25%
0
200
1000
2000
3000
4000
5000

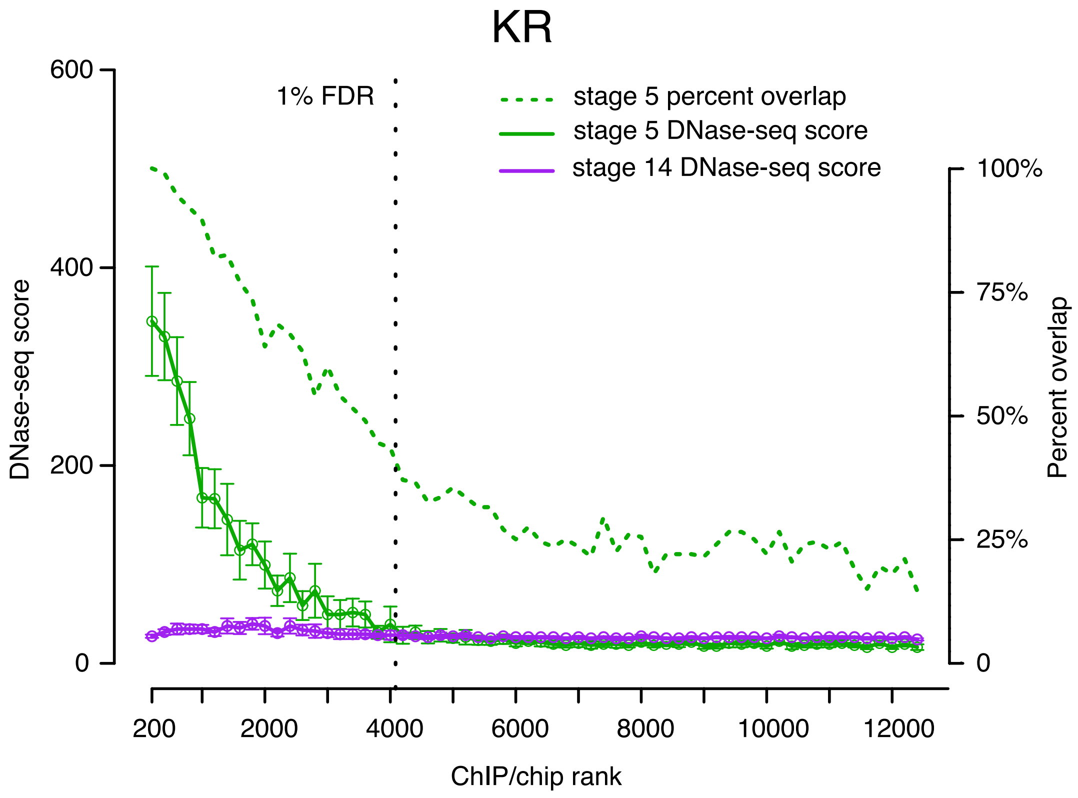

KR
1% FDR
stage 5 percent overlap
stage 5 DNase-seq score
stage 14 DNase-seq score
DNase-seq score
600
400
200
0
Percent overlap
100%
75%
50%
25%
0
200
2000
4000
6000
8000
10000
12000
ChIP/chip rank

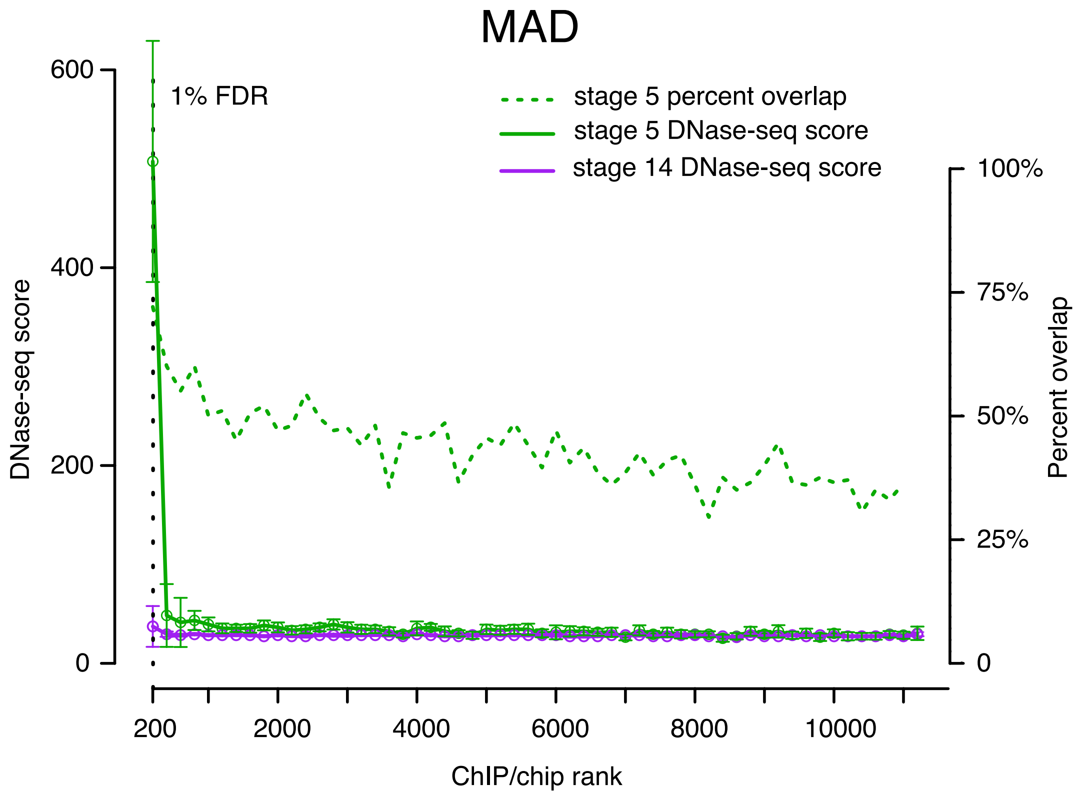

MAD
1% FDR
stage 5 percent overlap
stage 5 DNase-seq score
stage 14 DNase-seq score
DNase-seq score
0
200
400
600
Percent overlap
0
25%
50%
75%
100%
200
2000
4000
6000
8000
10000
ChIP/chip rank

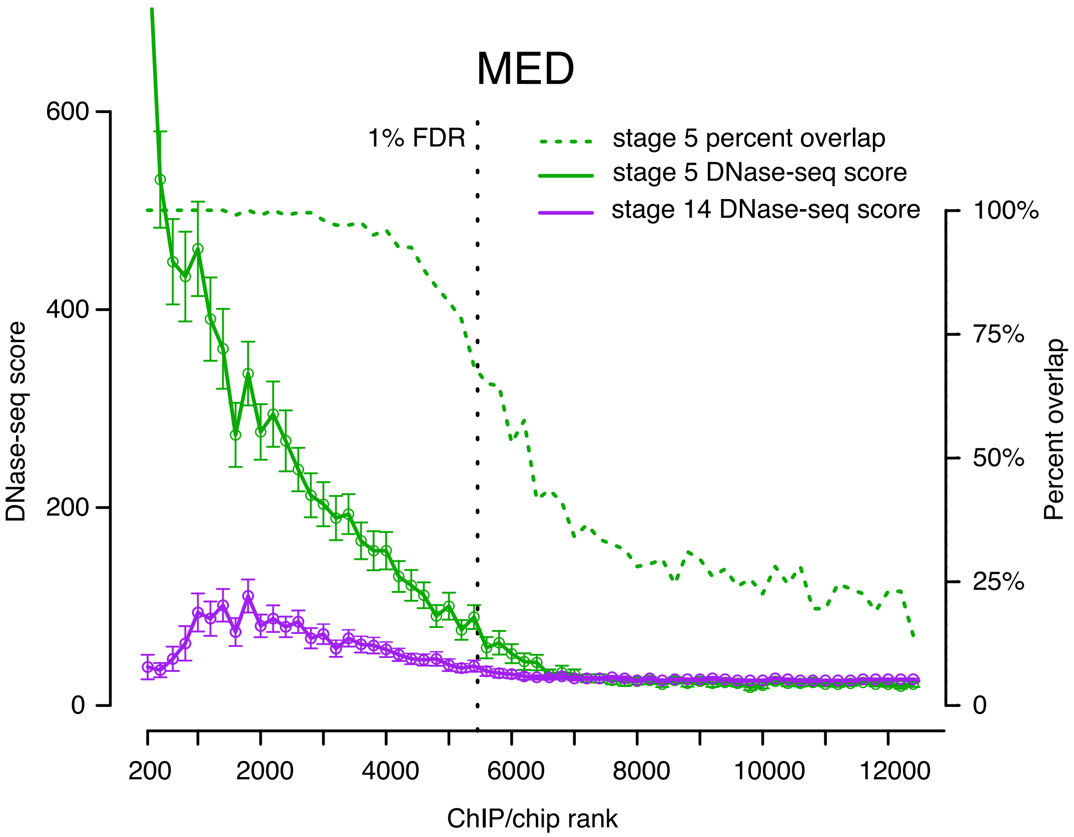
MED
1% FDR
stage 5 percent overlap
stage 5 DNase-seq score
stage 14 DNase-seq score
DNase-seq score
600
400
200
0
Percent overlap
100%
75%
50%
25%
0
200
2000
4000
6000
8000
10000
12000
ChIP/chip rank

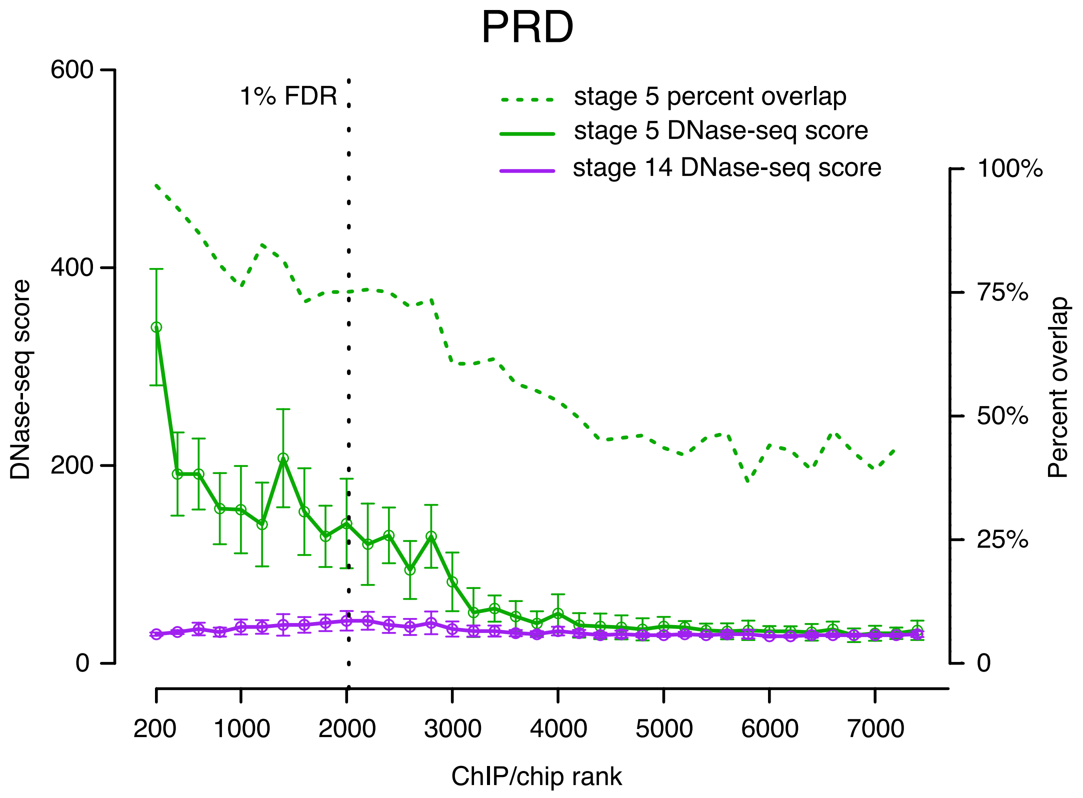

PRD
1% FDR
stage 5 percent overlap
stage 5 DNase-seq score
stage 14 DNase-seq score
DNase-seq score
Percent overlap
ChIP/chip rank
0
200
400
600
0
25%
50%
75%
100%
200
1000
2000
3000
4000
5000
6000
7000

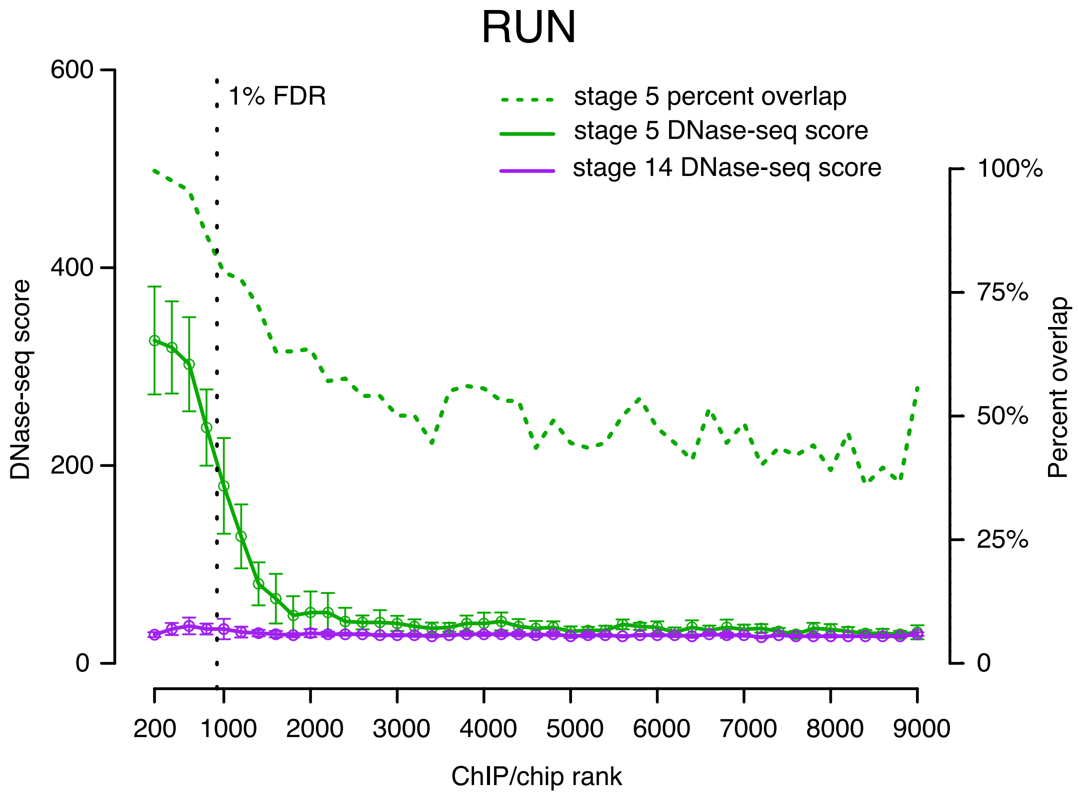
RUN
600
400
200
0
DNase-seq score
1% FDR
stage 5 percent overlap
stage 5 DNase-seq score
stage 14 DNase-seq score
100%
75%
50%
25%
0
Percent overlap
200
1000
2000
3000
4000
5000
6000
7000
8000
9000
ChIP/chip rank

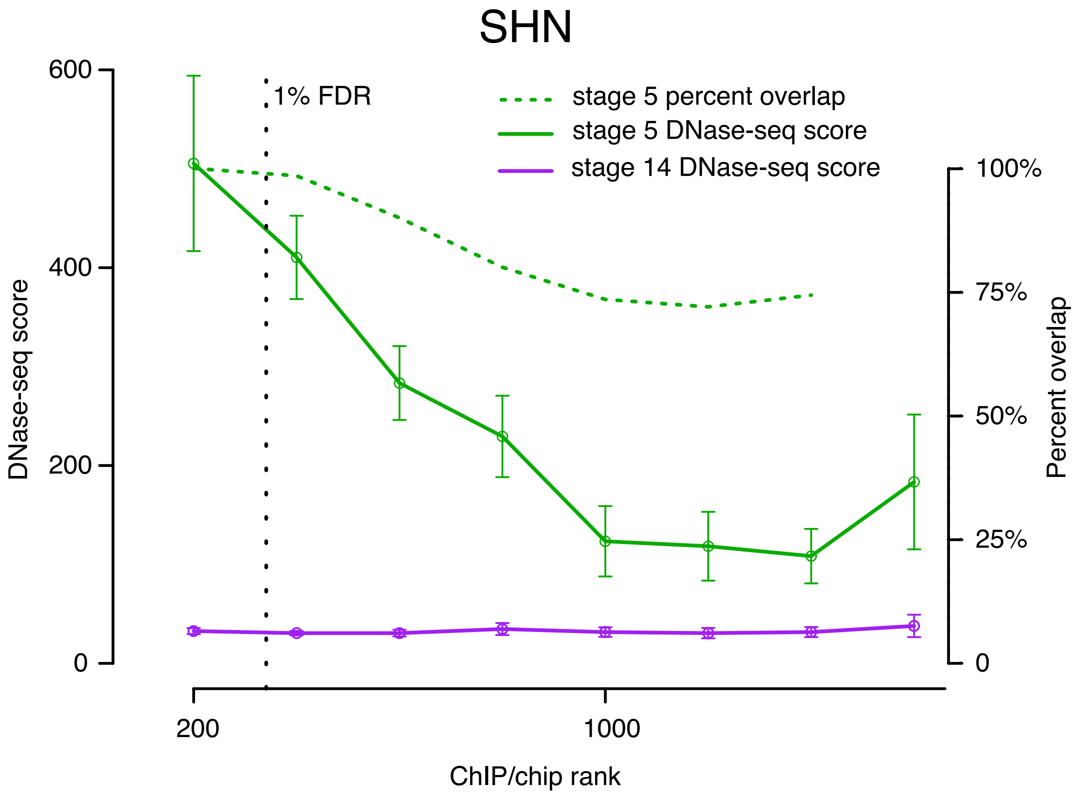
SHN
1% FDR
stage 5 percent overlap
stage 5 DNase-seq score
stage 14 DNase-seq score
600
400
200
0
DNase-seq score
100%
75%
50%
25%
0
Percent overlap
200
1000
ChIP/chip rank

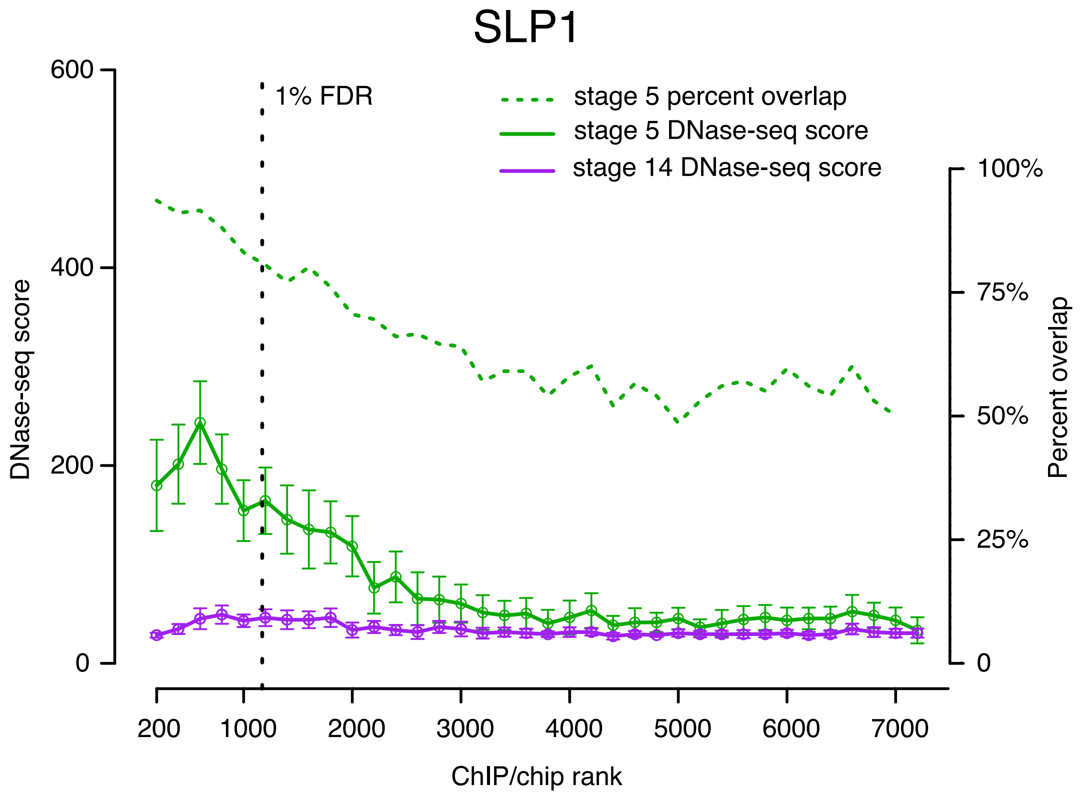

SLP1
1% FDR
stage 5 percent overlap
stage 5 DNase-seq score
stage 14 DNase-seq score
DNase-seq score
600
400
200
0
Percent overlap
100%
75%
50%
25%
0
200
1000
2000
3000
4000
5000
6000
7000
ChIP/chip rank

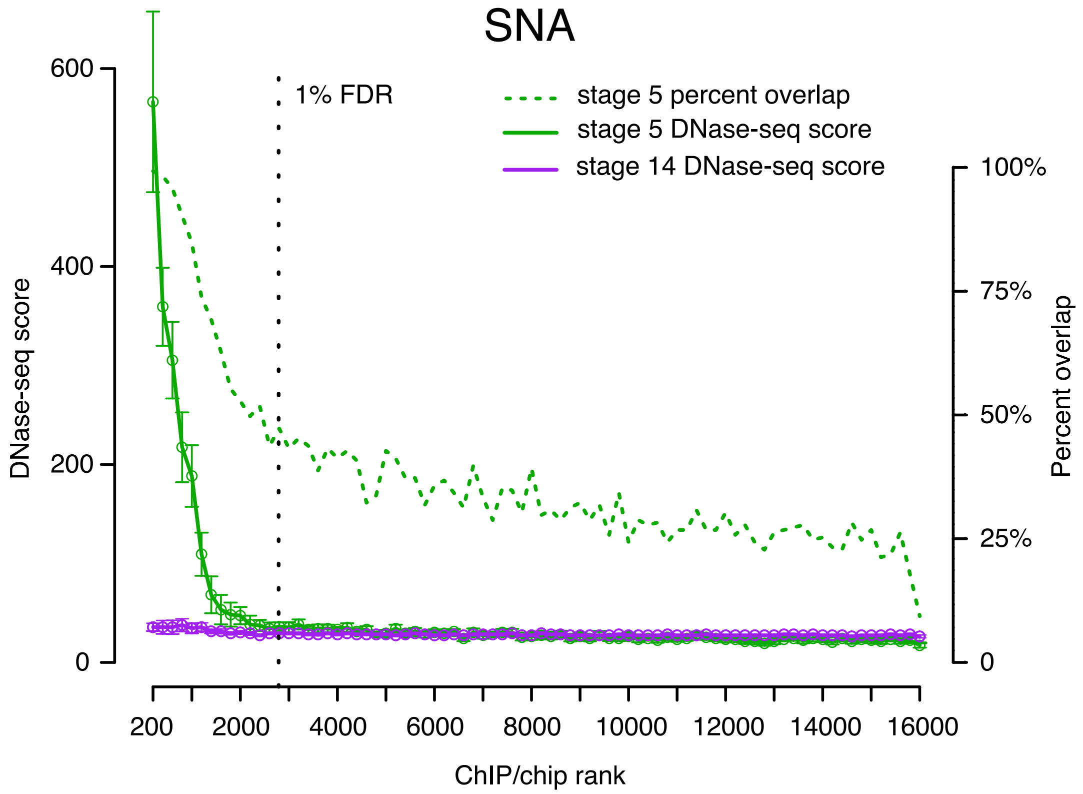

SNA
1% FDR
stage 5 percent overlap
stage 5 DNase-seq score
stage 14 DNase-seq score
DNase-seq score
600
400
200
0
100%
75%
50%
25%
0
Percent overlap
200
2000
4000
6000
8000
10000
12000
14000
16000
ChIP/chip rank

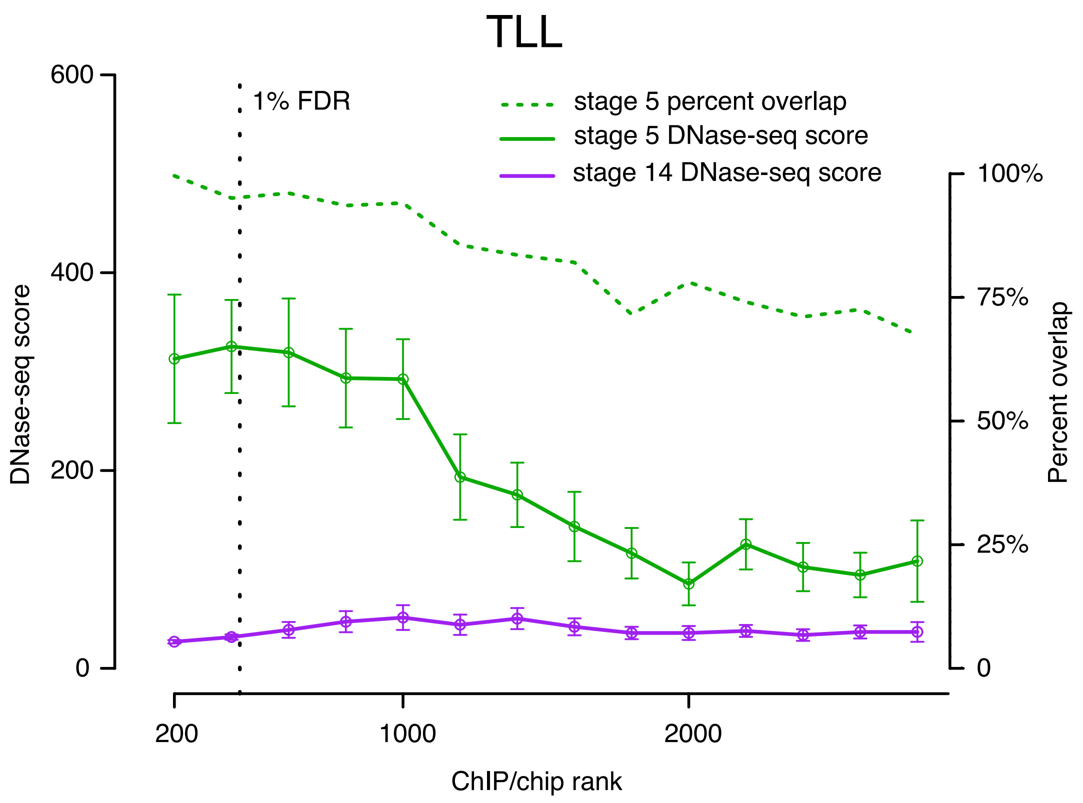
TLL
600
400
200
0
DNase-seq score
1% FDR
stage 5 percent overlap
stage 5 DNase-seq score
stage 14 DNase-seq score
100%
75%
50%
25%
0
Percent overlap
200
1000
2000
ChIP/chip rank

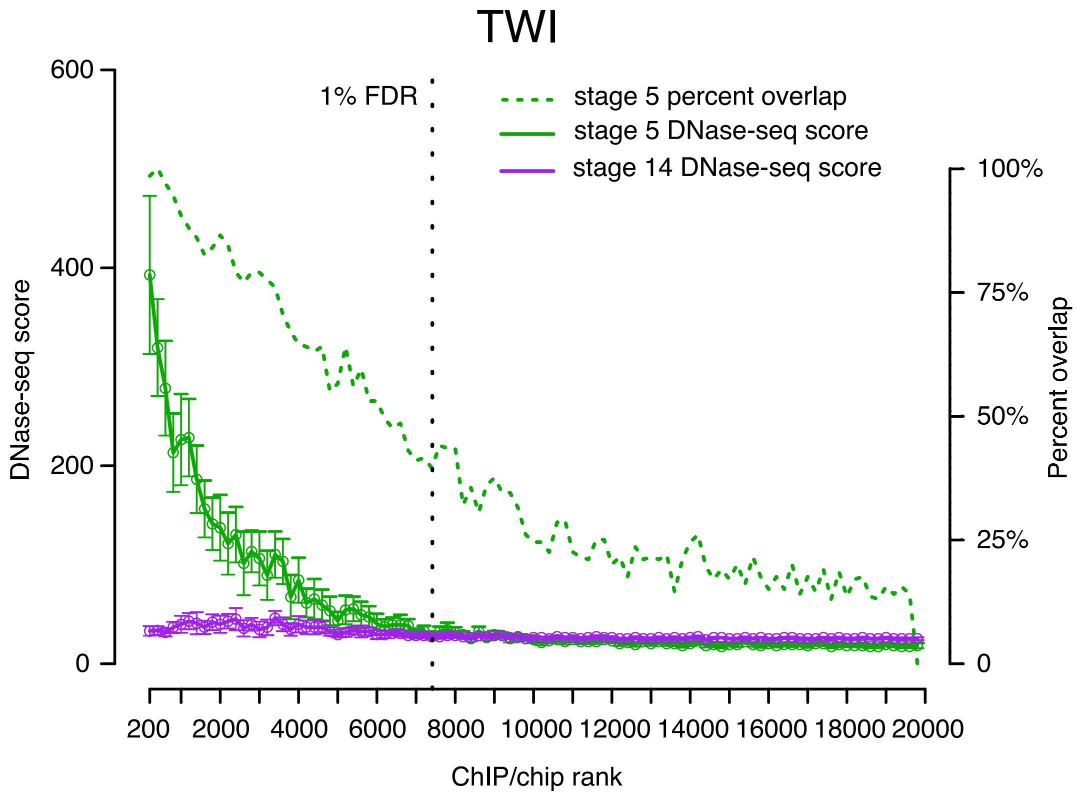
TWI
1% FDR
stage 5 percent overlap
stage 5 DNase-seq score
stage 14 DNase-seq score
DNase-seq score
600
400
200
0
100%
75%
50%
25%
0
Percent overlap
200
2000
4000
6000
8000
10000
12000
14000
16000
18000
20000
ChIP/chip rank
