# Supplementary material for: The role of chromatin accessibility in directing the widespread, overlapping patterns of Drosophila transcription factor binding
Source: Genome Biol. 2011 Apr 7;12(4):R34. doi: 10.1186/gb-2011-12-4-r34 (PMC3218860; doi:10.1186/gb-2011-12-4-r34)
Supplement: Additional file 8 — Comparison of ChIP-chip scores for occurrences of DNA recognition sequences in accessible versus closed chromatin regions. [file gb-2011-12-4-r34-S8.PDF]

Additional data file 8. **Comparison of ChIP-chip scores for occurrences of DNA recognition sequences in accessible versus closed chromatin regions.** Separately for each transcription factor, all significant recognition sequences in the euchromatic genome for four affinity cohorts were identified using PWMs derived from *in vitro* DNA binding data (Table 1) [17,70]. In addition, for each factor sequence matches to ten PWMs derived by random permutation of nucleotide position order were derived. DNA sites in each affinity cohort for both the genuine and scrambled PWMs present were each classified as either accessible or inaccessible, using the 5% FDR DNase accessible regions to define accessible regions (Table 1). The median ChIP-chip scores (y axis) for the 500 bp regions +/- 250 bp around recognition sites in each affinity cohort were plotted separately for accessible (red lines) and inaccessible (blue lines) genomic regions. Dark red and blue lines show results for the genuine factor PWMs, light red and blue lines the median result for the scrambled PWMs. The highest affinity cohort is to the left (x axis). Web logo representations of the genuine PWM representing the highest affinity cohort and the lowest affinity cohort of recognition sites are shown at the bottom. The 95% confidence limits for the median ChIP-chip scores are shown.

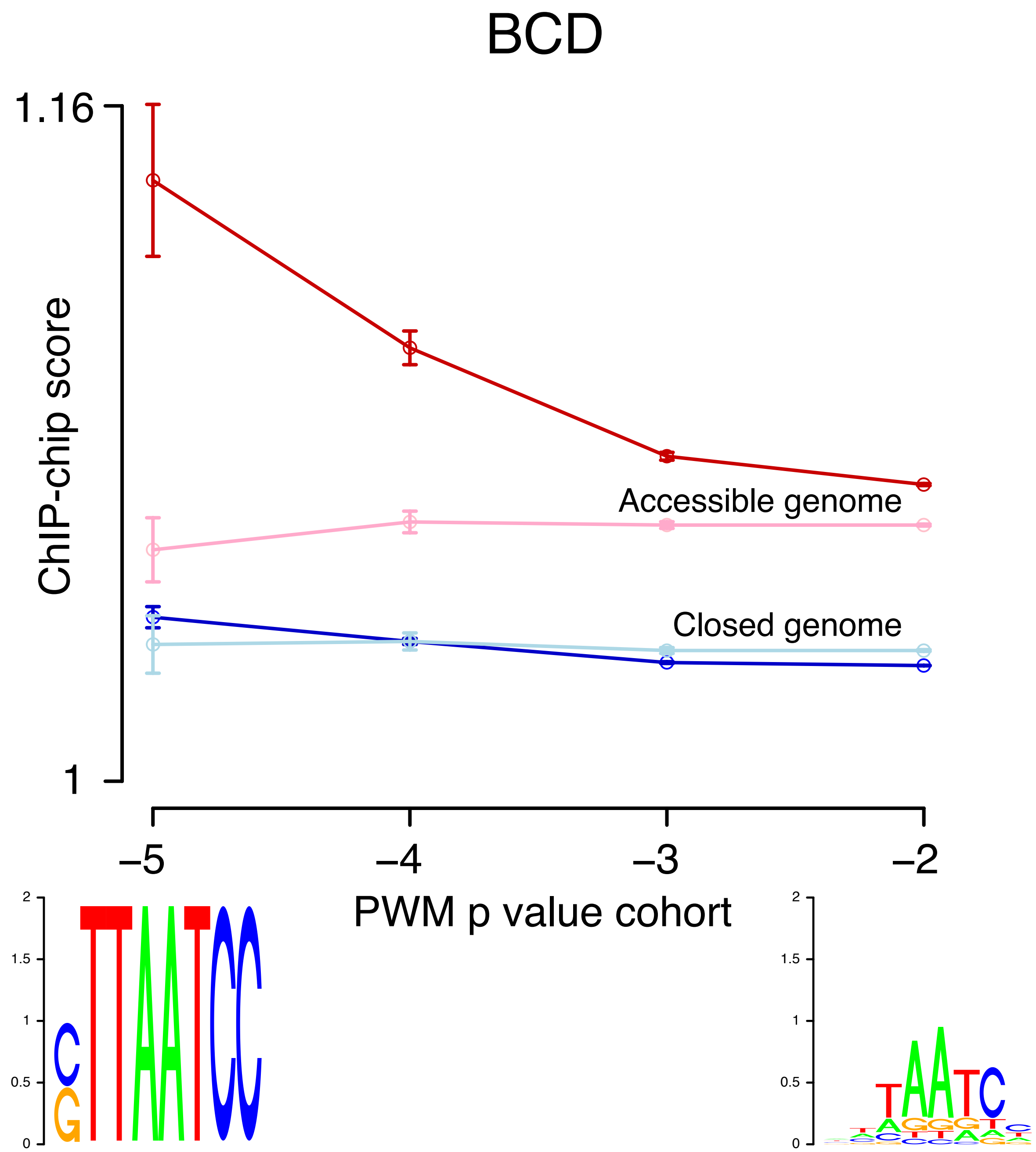
BCD
1.16
1
ChIP-chip score
Accessible genome
Closed genome
−5
−4
−3
−2
PWM p value cohort
2
1.5
1
0.5
0
2
1.5
1
0.5
0

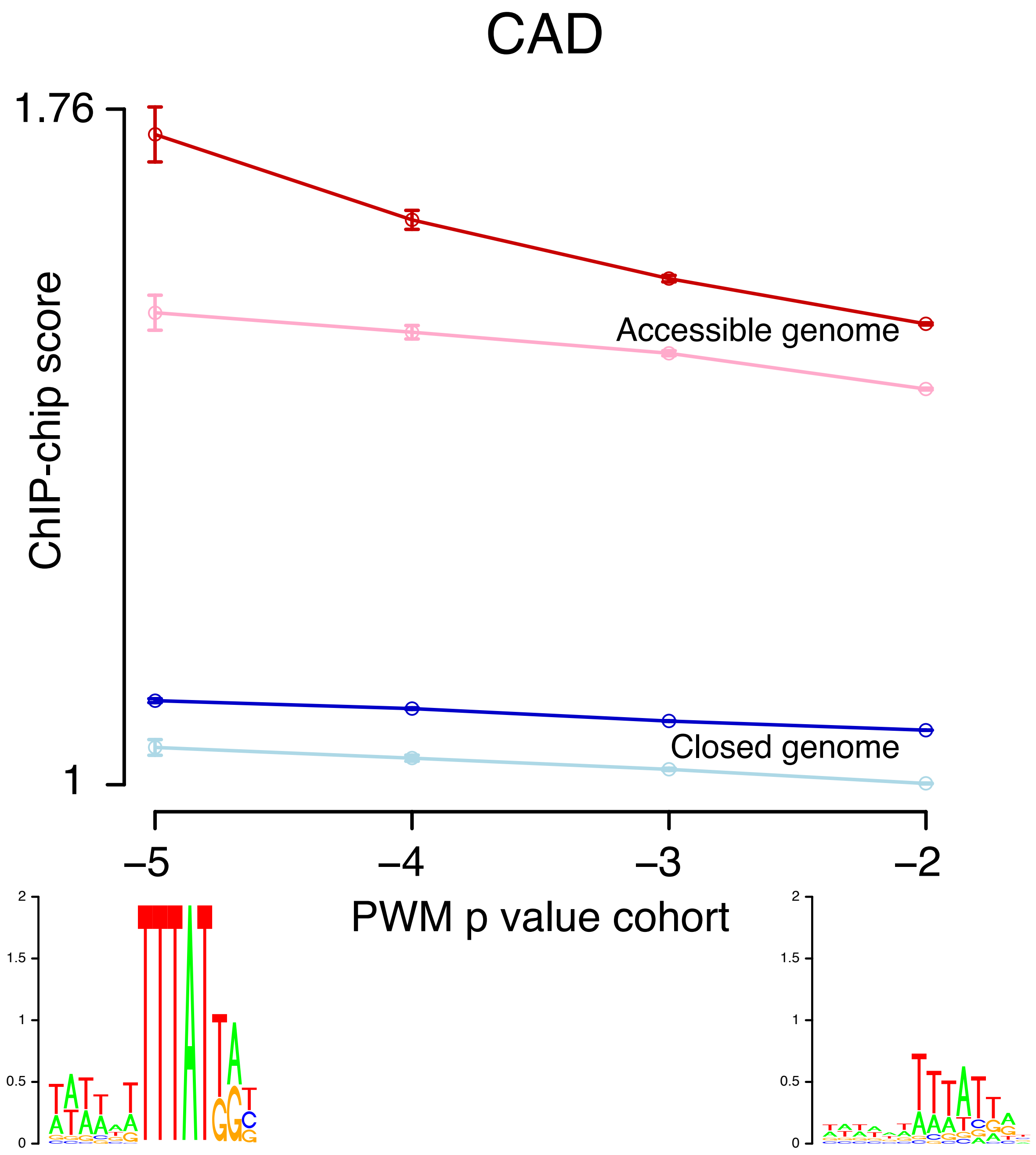

CAD
1.76
1
ChIP-chip score
Accessible genome
Closed genome
–5
–4
–3
–2
PWM p value cohort
2
1.5
1
0.5
0

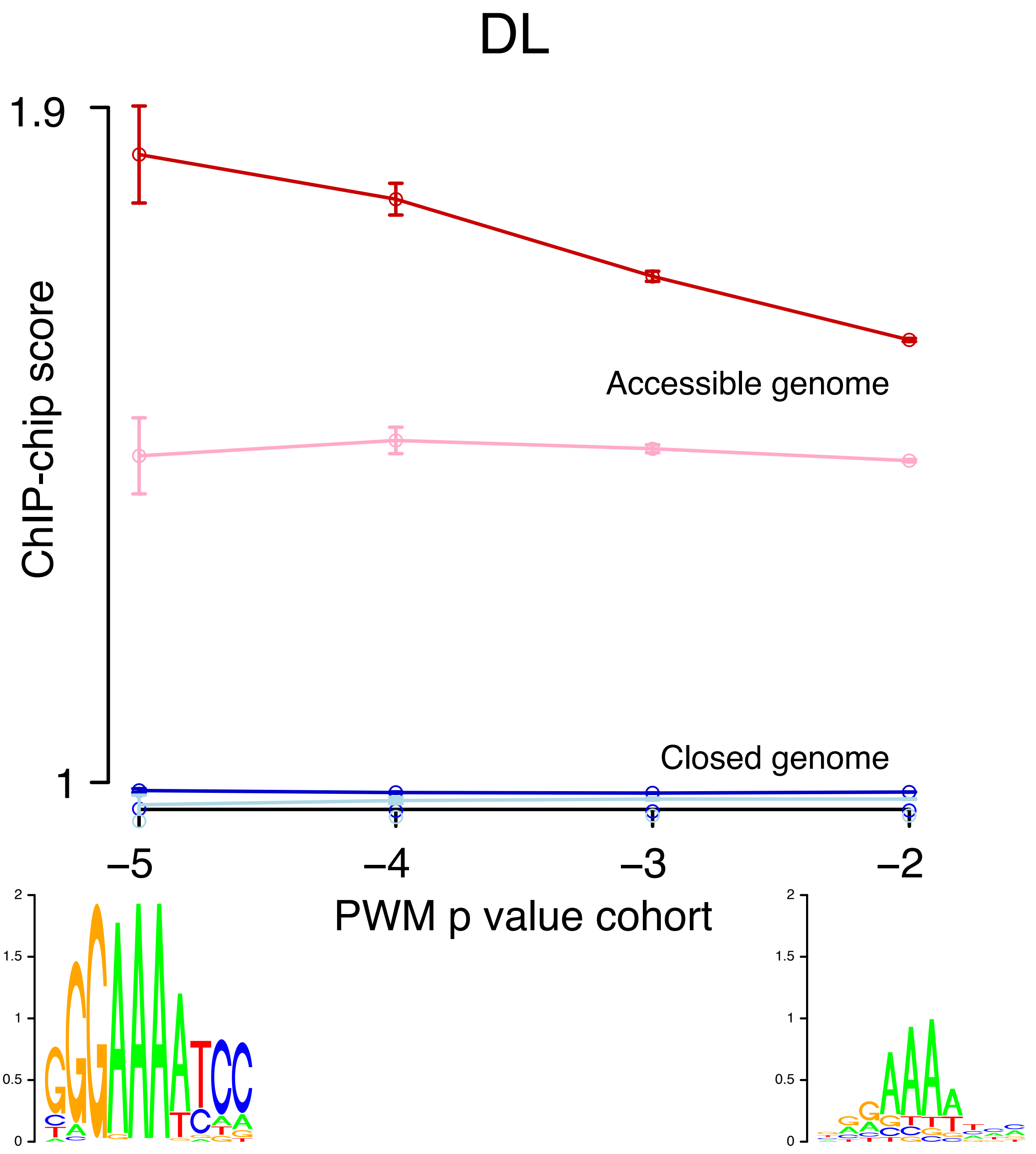
DL
ChIP-chip score
1.9
1
Accessible genome
Closed genome
−5
−4
−3
−2
PWM p value cohort
2
1.5
1
0.5
0

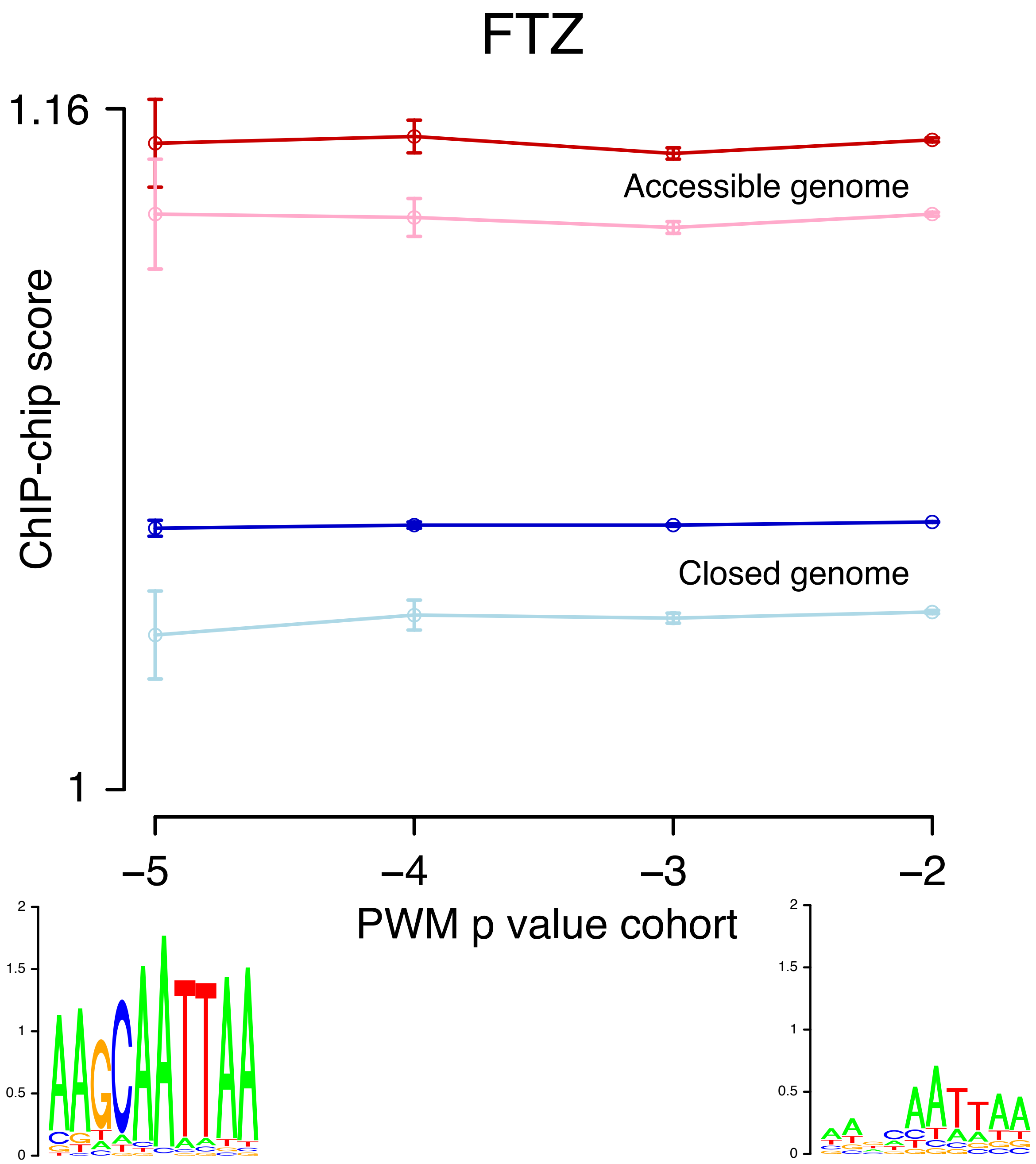

FTZ
1.16
1
ChIP-chip score
Accessible genome
Closed genome
–5
–4
–3
–2
PWM p value cohort
2
1.5
1
0.5
0

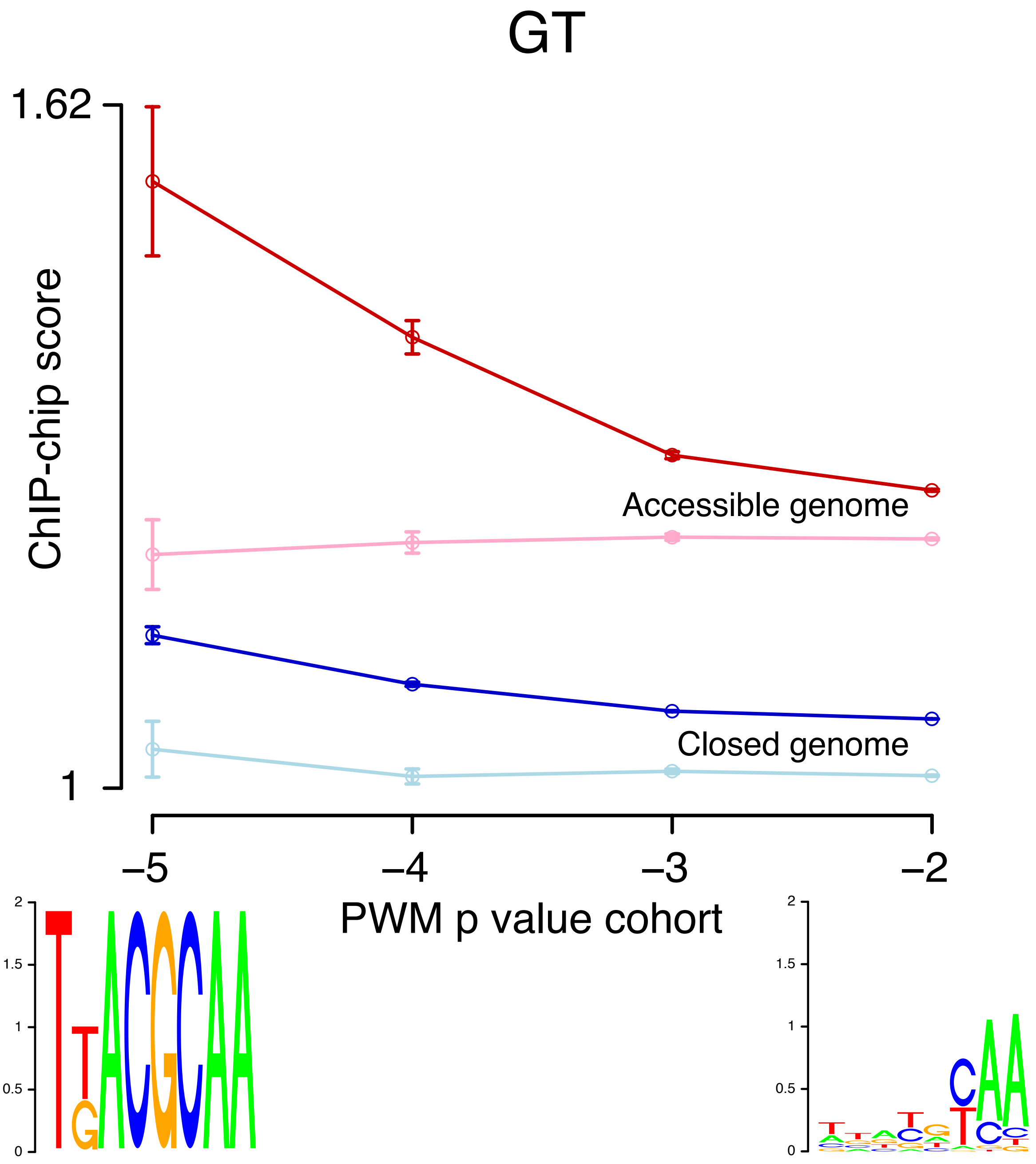

GT
1.62
1
ChIP-chip score
Accessible genome
Closed genome
−5
−4
−3
−2
PWM p value cohort
2
1.5
1
0.5
0

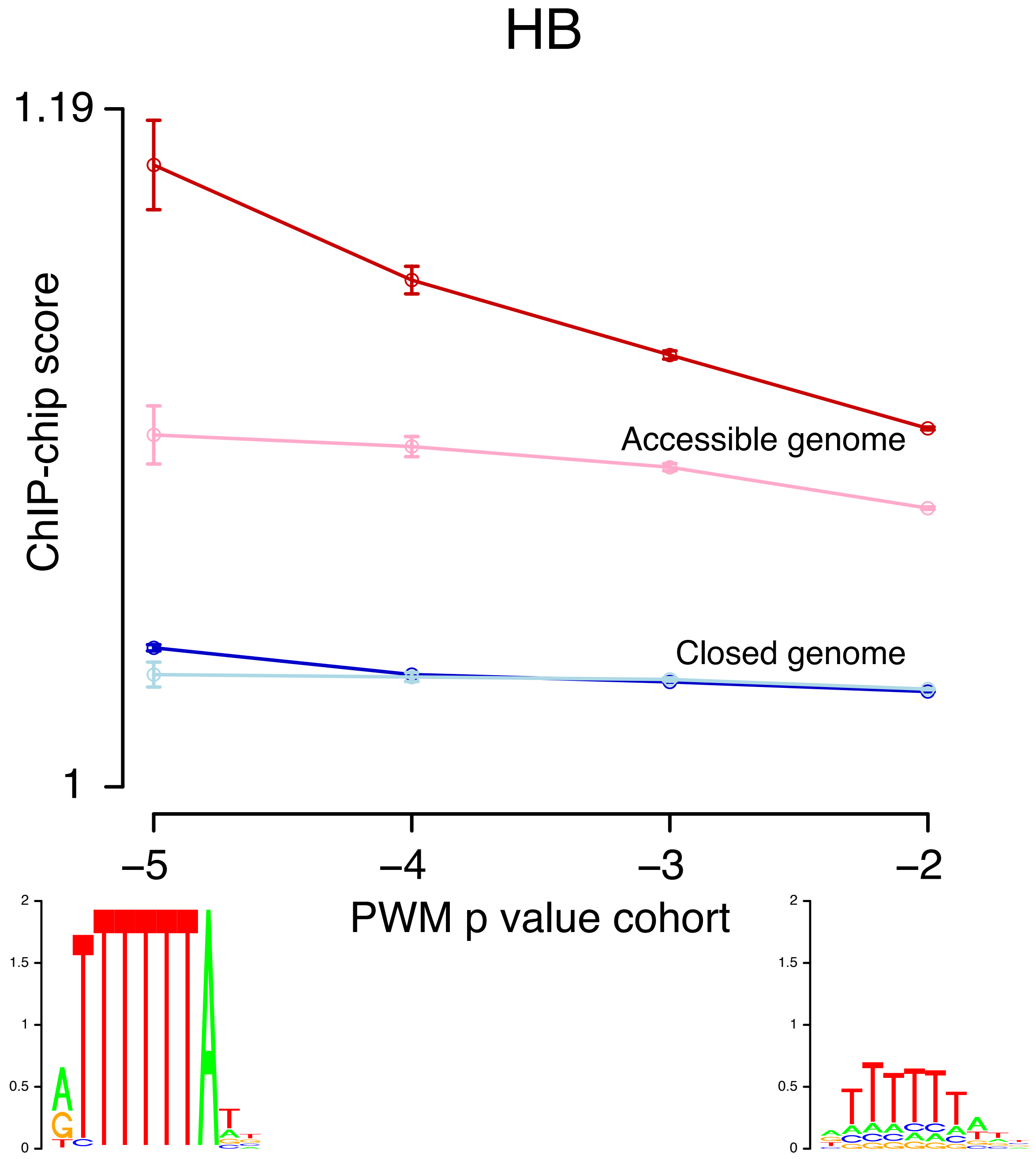
HB
1.19
1
ChIP-chip score
Accessible genome
Closed genome
−5
−4
−3
−2
PWM p value cohort
2
1.5
1
0.5
0

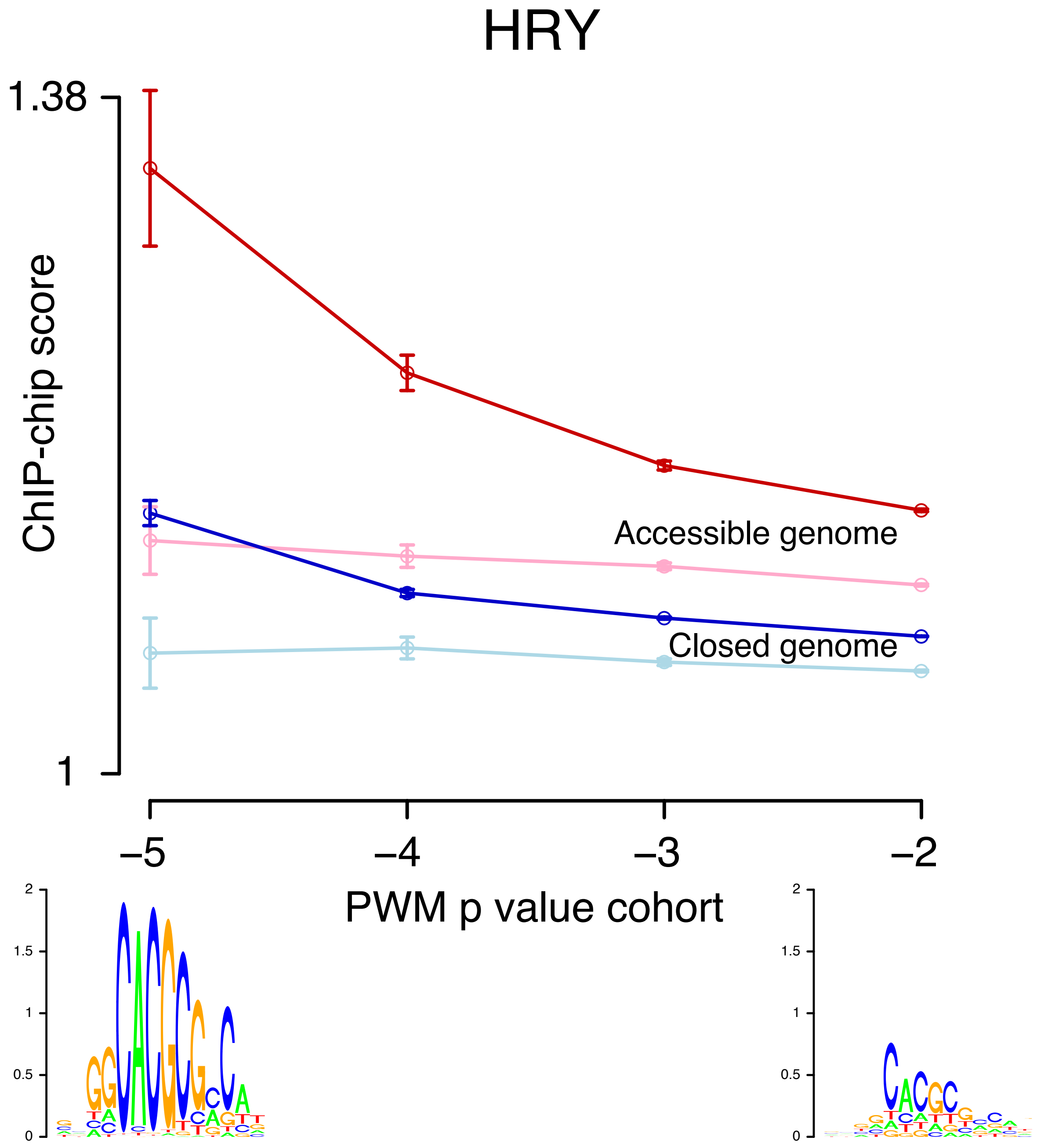
HRY
1.38
1
ChIP-chip score
Accessible genome
Closed genome
−5
−4
−3
−2
PWM p value cohort
2
1.5
1
0.5
0

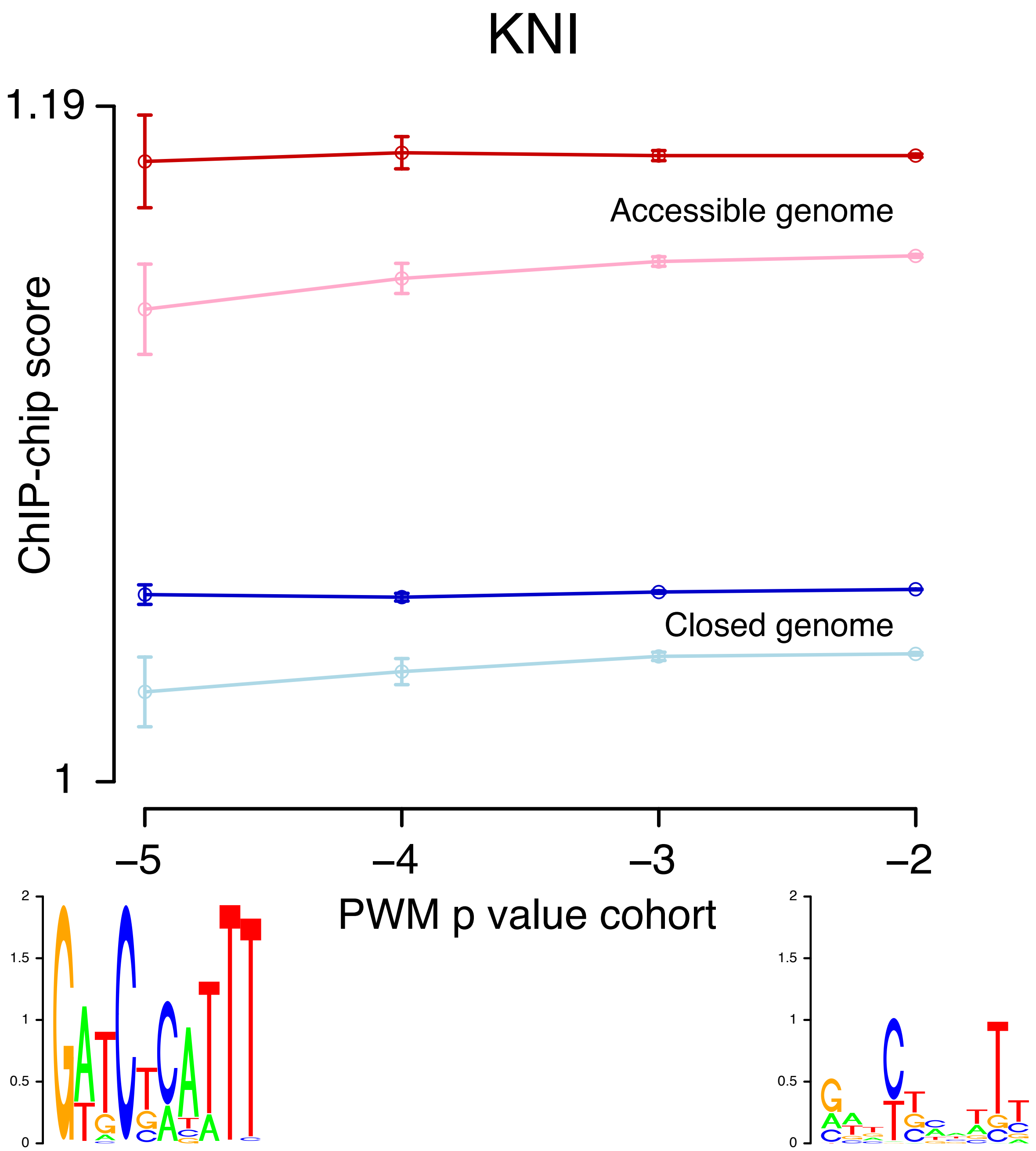
KNI
1.19
1
ChIP-chip score
Accessible genome
Closed genome
−5
−4
−3
−2
PWM p value cohort
2
1.5
1
0.5
0

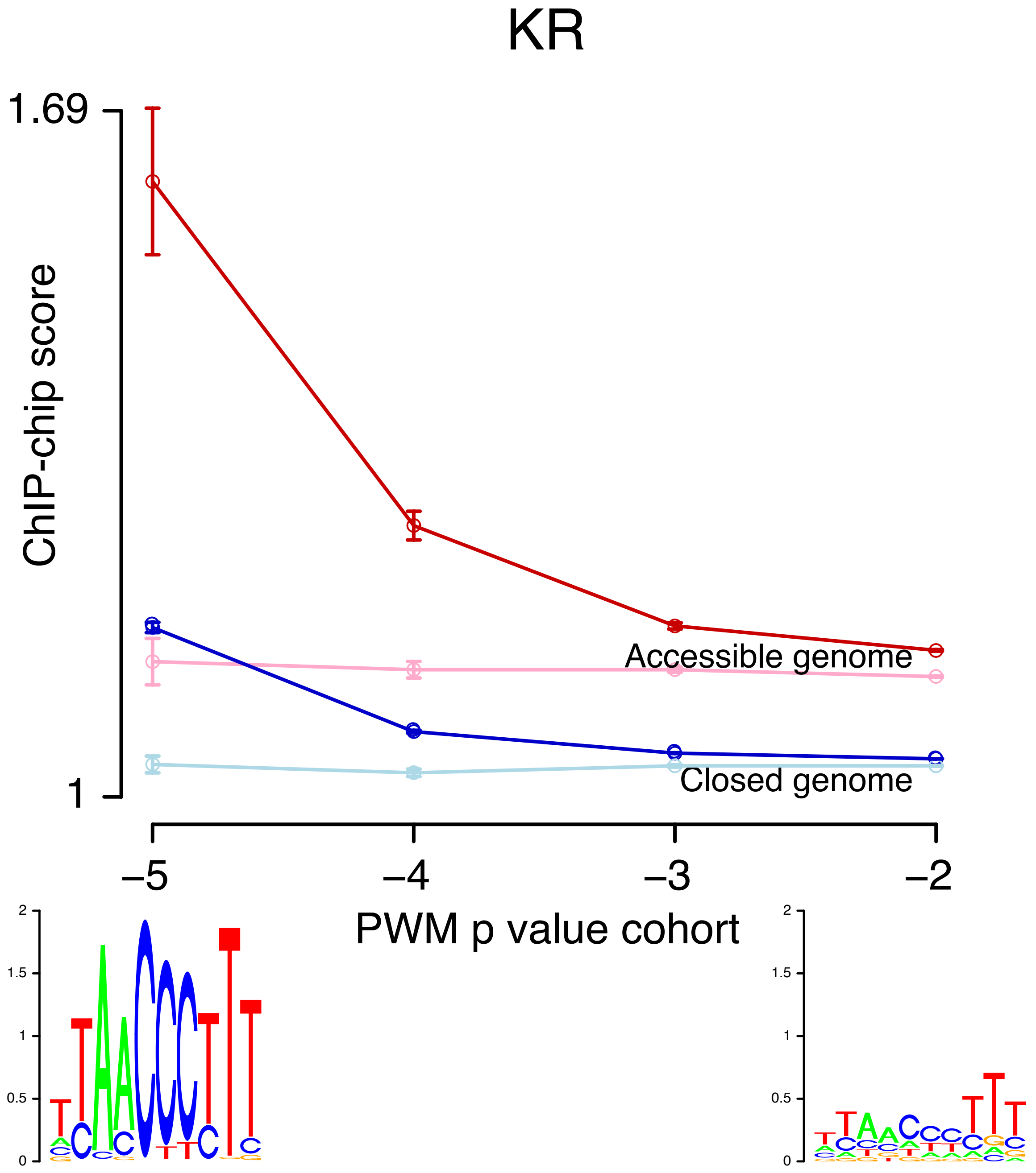

KR
1.69
1
ChIP-chip score
Accessible genome
Closed genome
−5
−4
−3
−2
PWM p value cohort

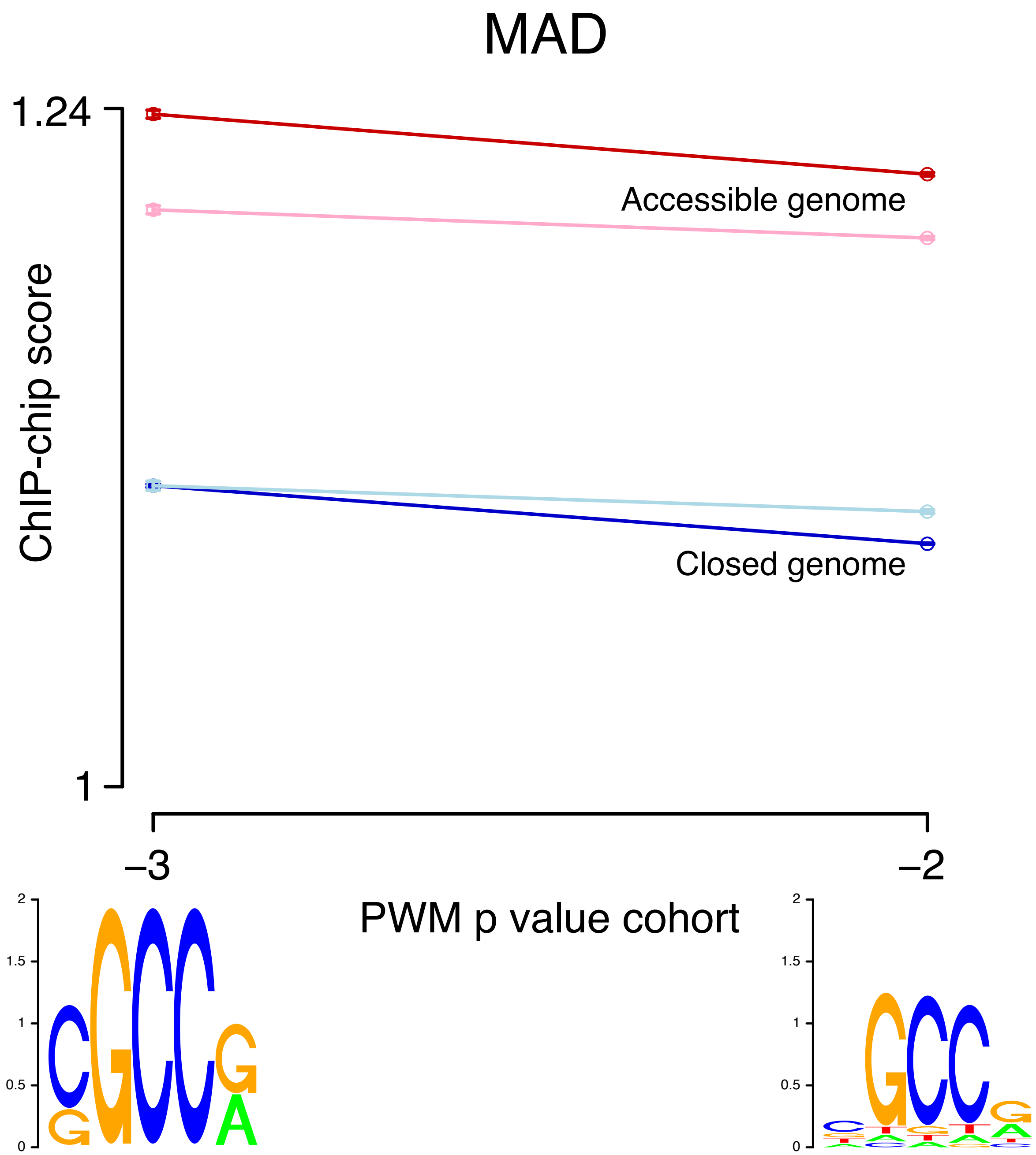

MAD
1.24
1
ChIP-chip score
Accessible genome
Closed genome
−3
−2
PWM p value cohort
2
1.5
1
0.5
0

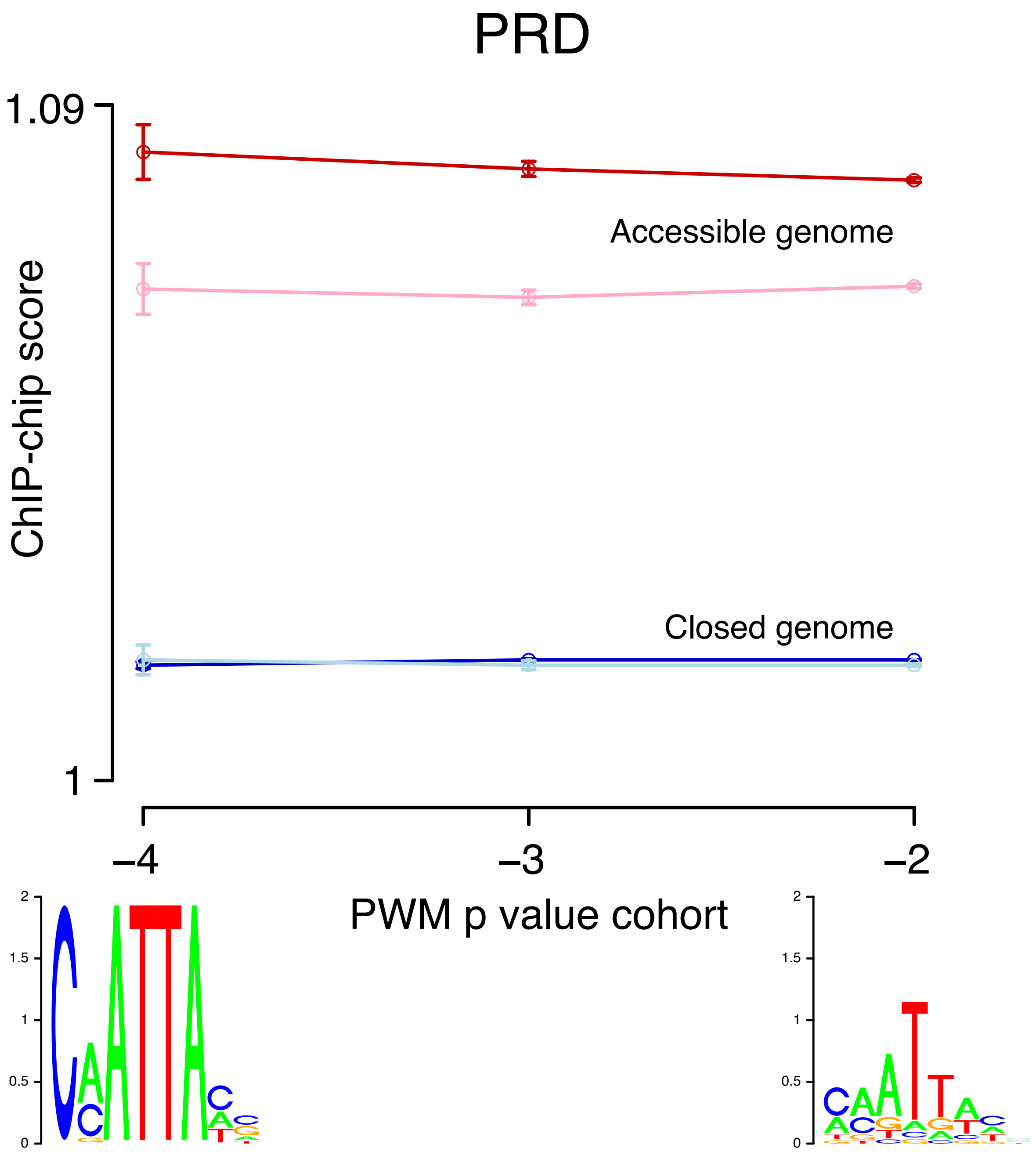

PRD
1.09
1
ChIP-chip score
Accessible genome
Closed genome
−4
−3
−2
PWM p value cohort
2
1.5
1
0.5
0

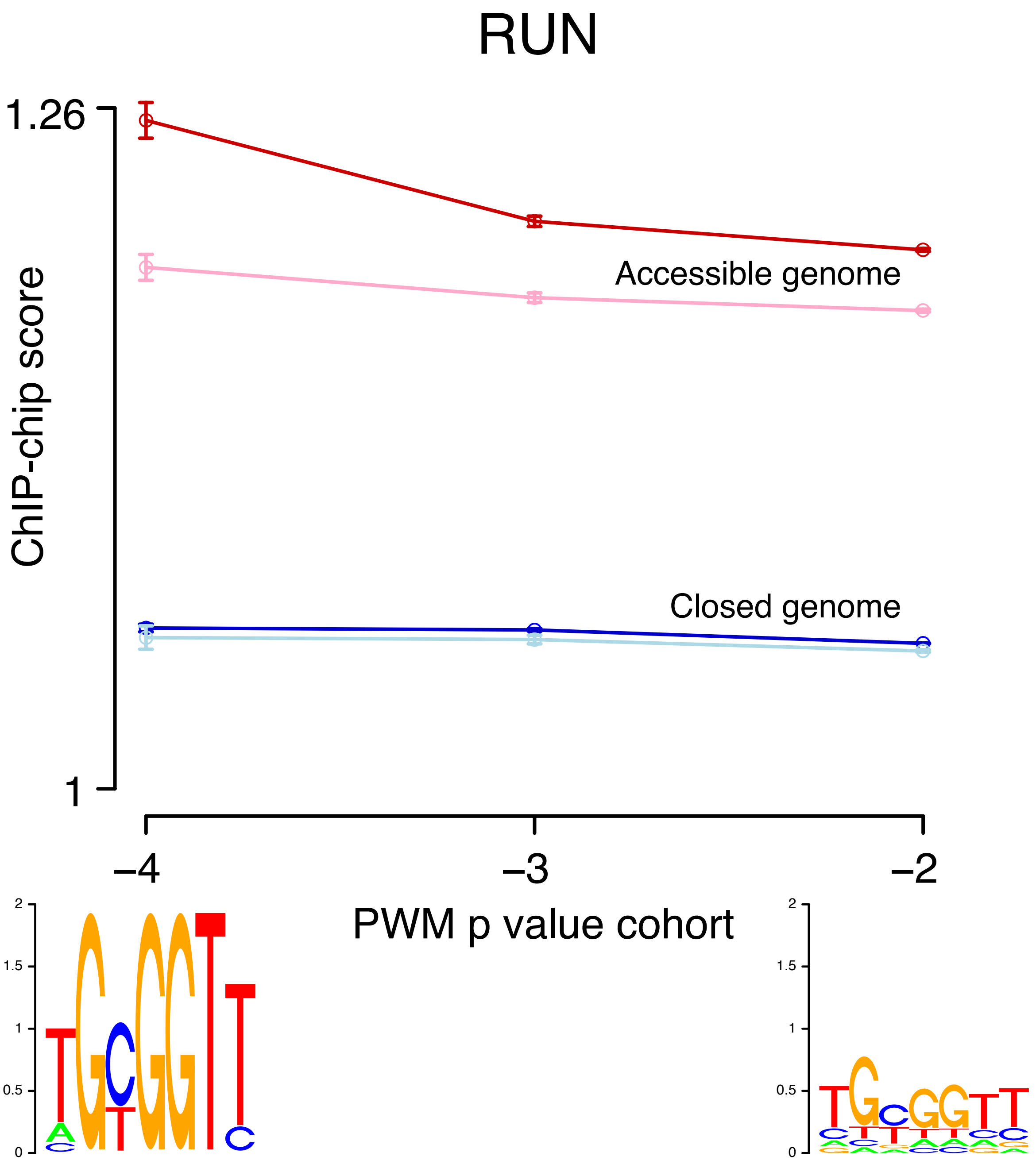
RUN
1.26
1
ChIP-chip score
Accessible genome
Closed genome
−4
−3
−2
PWM p value cohort
2
1.5
1
0.5
0

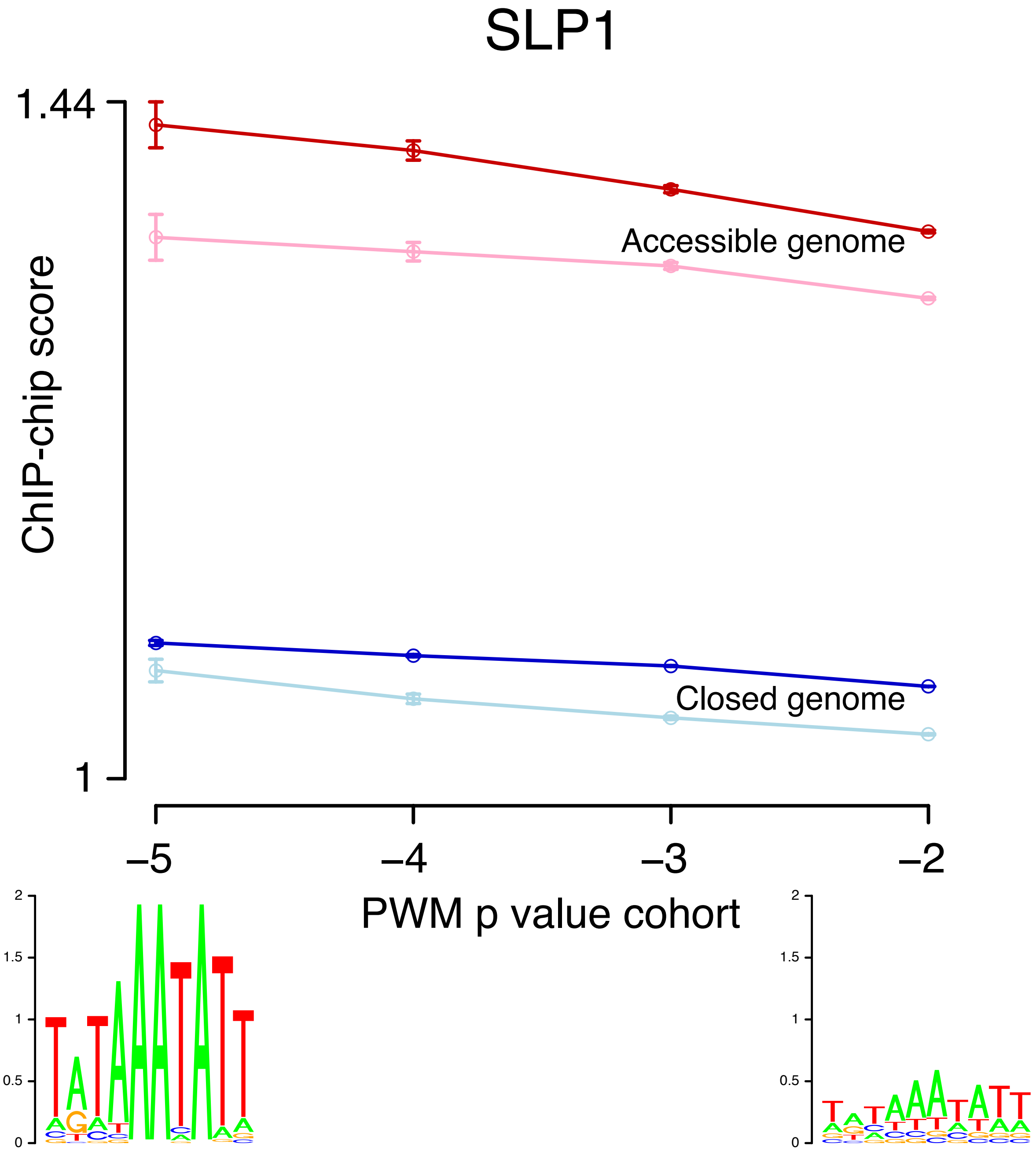

SLP1
1.44
1
ChIP-chip score
Accessible genome
Closed genome
−5
−4
−3
−2
PWM p value cohort
2
1.5
1
0.5
0

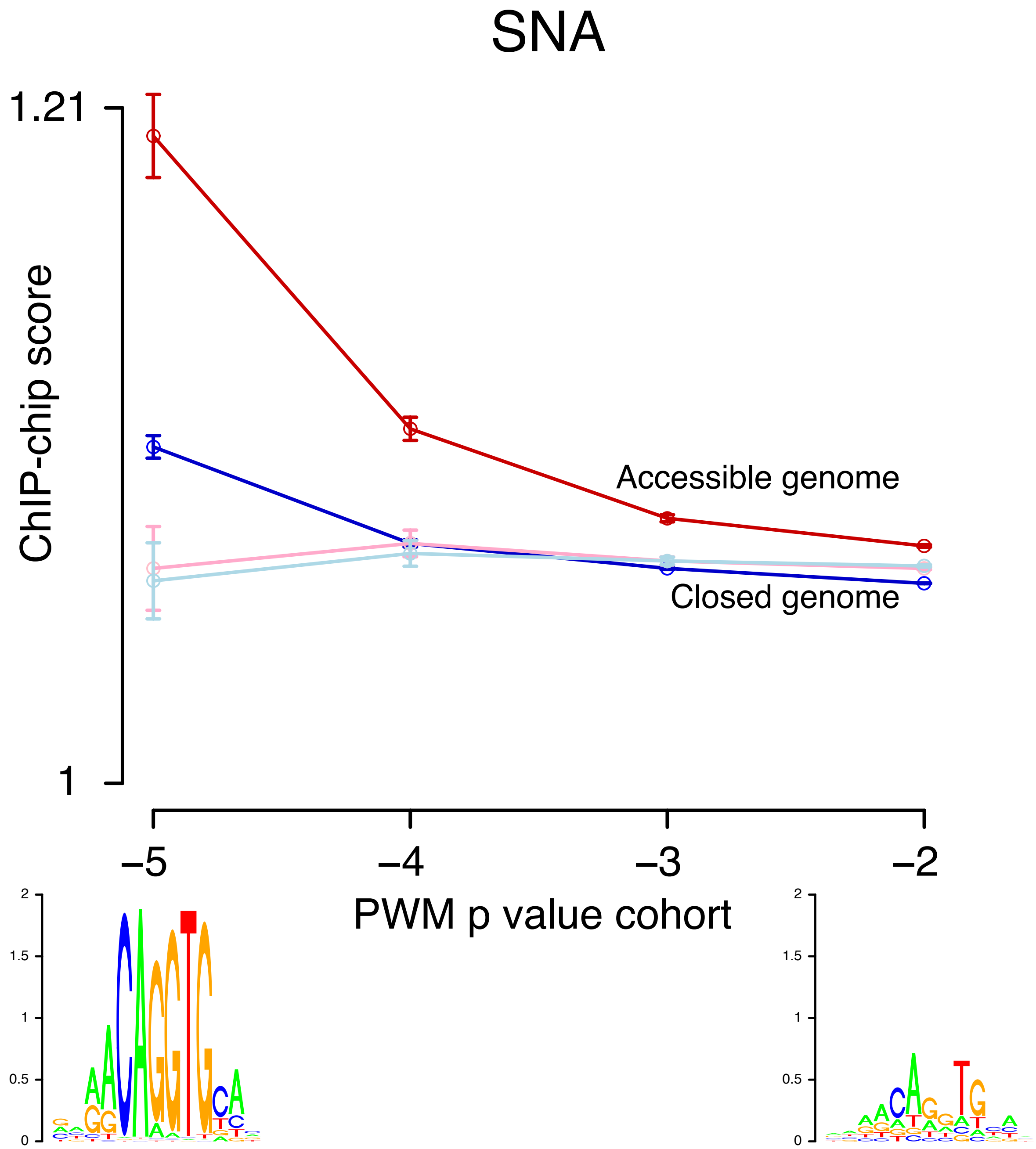
SNA
1.21
1
ChIP-chip score
Accessible genome
Closed genome
−5
−4
−3
−2
PWM p value cohort
2
1.5
1
0.5
0
2
1.5
1
0.5
0

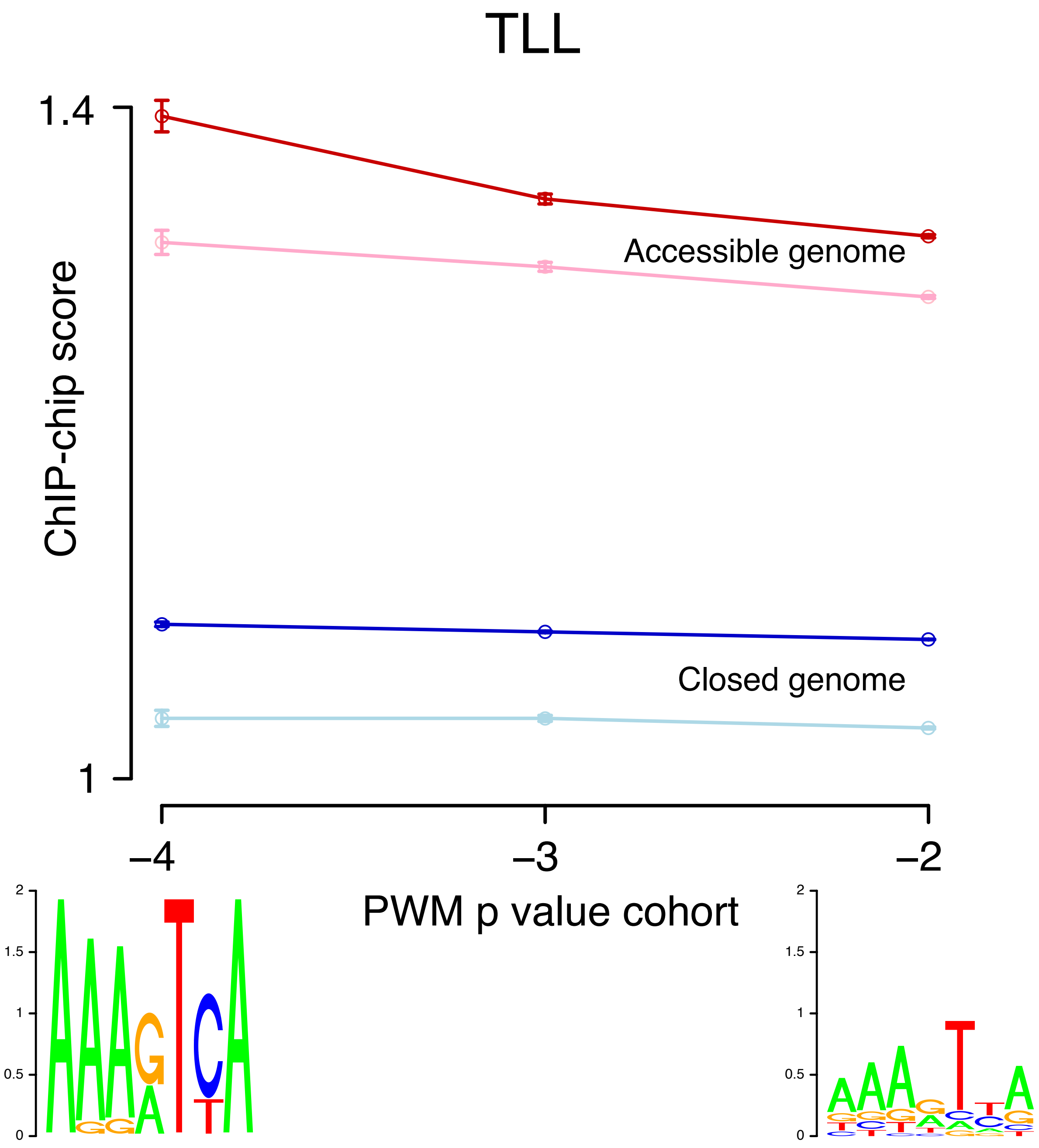
TLL
1.4
1
ChIP-chip score
Accessible genome
Closed genome
−4
−3
−2
PWM p value cohort
2
1.5
1
0.5
0

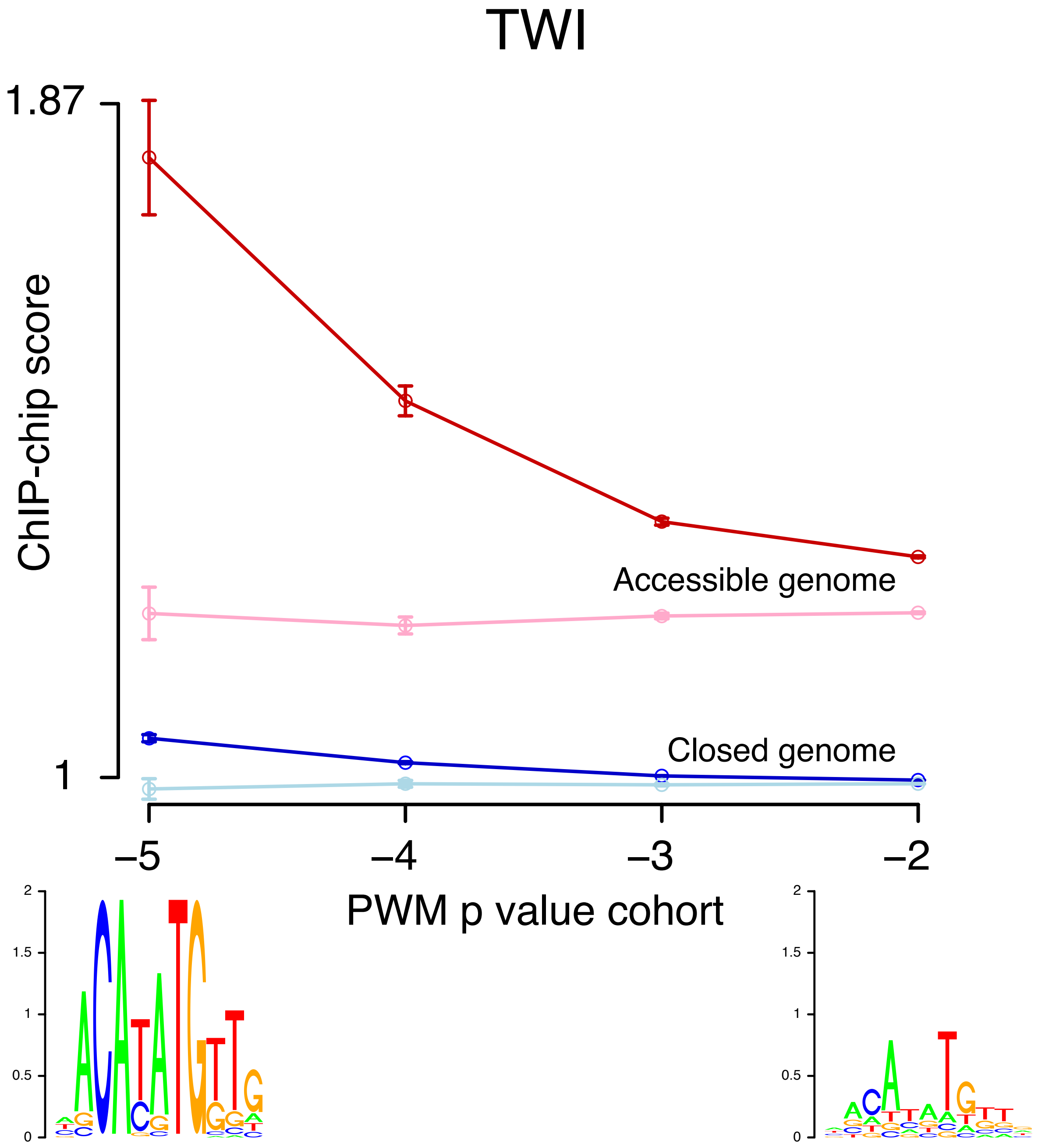
TWI
1.87
1
ChIP-chip score
Accessible genome
Closed genome
−5
−4
−3
−2
PWM p value cohort
2
1.5
1
0.5
0
