# Supplementary material for: The role of chromatin accessibility in directing the widespread, overlapping patterns of Drosophila transcription factor binding
Source: Genome Biol. 2011 Apr 7;12(4):R34. doi: 10.1186/gb-2011-12-4-r34 (PMC3218860; doi:10.1186/gb-2011-12-4-r34)

**Additional data file 9. Levels of MED factor occupancy and DNaseI accessibility change between developmental stages.** The level of MED binding and DNaseI accessibility on the scnf (left), dpp (center), and shn (right) genes are shown at stages 5, 10 and 14. The figure is labeled using the same conventions in Figure 1 except that the locations of the regions above the ChIP-chip 1% FDR threshold are indicated by black horizontal lines beneath the continuous traces of ChIP-chip scores.

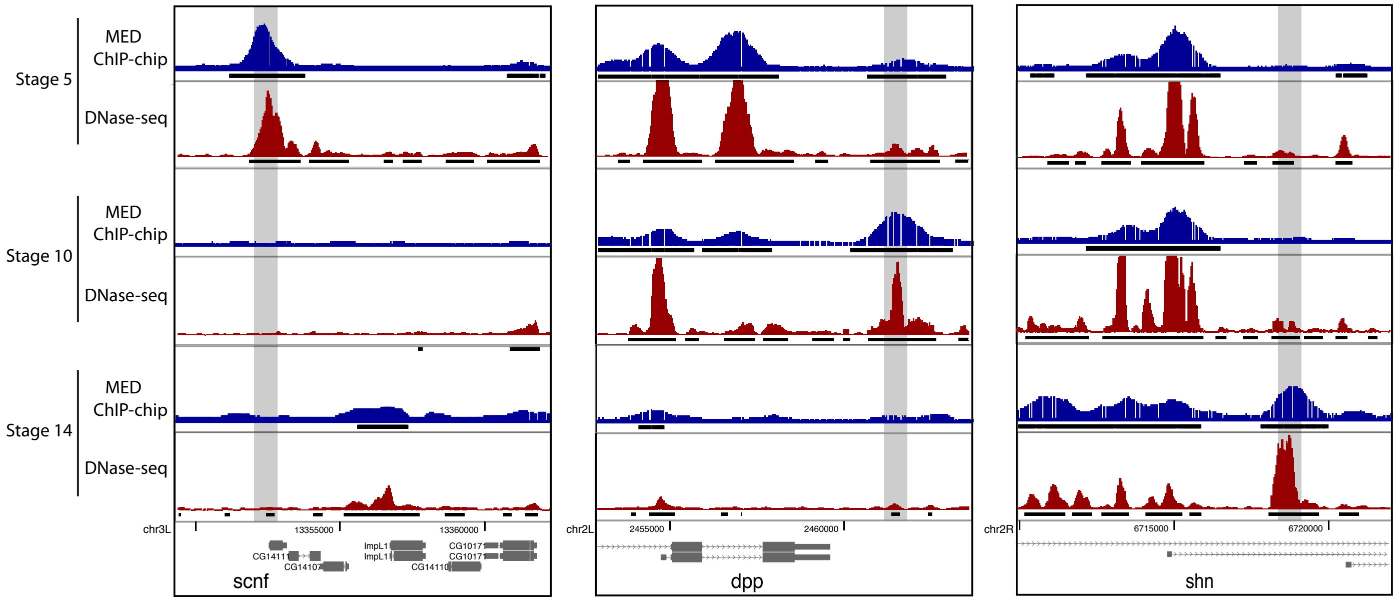

Supplement: Additional file 9 — Levels of MED factor occupancy and DNaseI accessibility change between developmental stages. [file gb-2011-12-4-r34-S9.PDF]
