# Supplementary material for: The role of chromatin accessibility in directing the widespread, overlapping patterns of Drosophila transcription factor binding
Source: Genome Biol. 2011 Apr 7;12(4):R34. doi: 10.1186/gb-2011-12-4-r34 (PMC3218860; doi:10.1186/gb-2011-12-4-r34)

Additional data file 10. Change in DNA binding levels in vivo between developmental stages. The scatter plots take the ChIP-chip scores for the 1 kb regions +/- 500 bp of the peak nucleotide of binding for all 25% FDR peaks for the experiment plotted on the x-axis and compares those to the identical 1 kb regions from the experiment plotted on the y axis. **(A)** compares two experiments using antibodies against non overlapping portions of the HB protein at stage 5. **(B)** compares binding of HB at stages 5 and 9. **(C)** compares binding of MED at stages 5 and stage 10. **(D)** compares binding of MED at stages 5 and 14. The Pearson correlation coefficients (r) for each comparison are show above each panel.

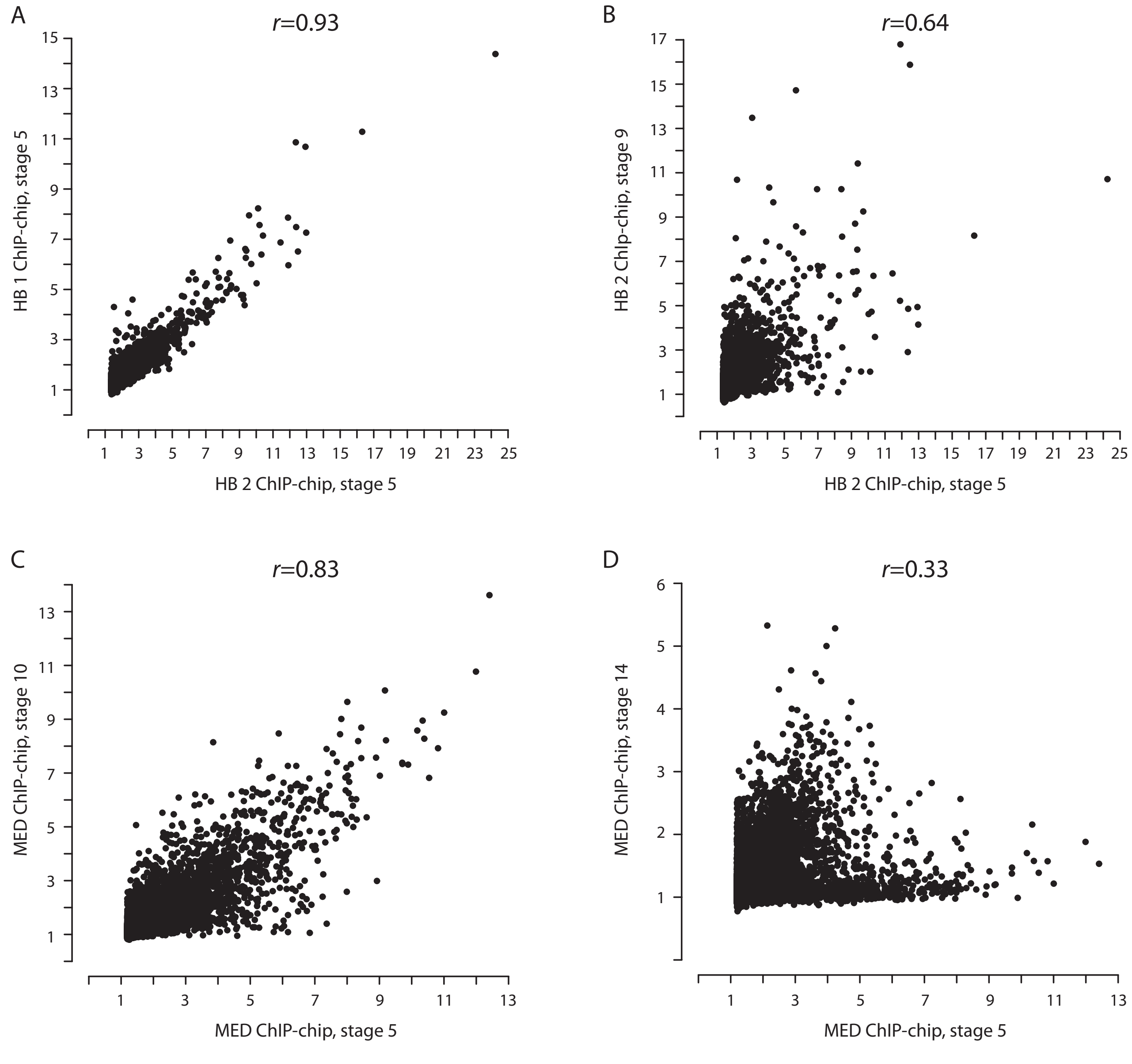

Supplement: Additional file 10 — Change in DNA binding levels in vivo between developmental stages. [file gb-2011-12-4-r34-S10.PDF]
