# Supplementary material for: The role of chromatin accessibility in directing the widespread, overlapping patterns of Drosophila transcription factor binding
Source: Genome Biol. 2011 Apr 7;12(4):R34. doi: 10.1186/gb-2011-12-4-r34 (PMC3218860; doi:10.1186/gb-2011-12-4-r34)

Additional data file 11. Temporal changes in levels of MED occupancy correlate with changes in DNaseI accessibility. 1 kb regions +/- 500 bp of the peak nucleotide of binding for each of the 400 regions most highly bound by MED at stages 5 (A and B), 10 (C) and 14 (D) were identified. (A) Plot of the ratio of ChIP-chip scores at stage 5 over those at 10 (x axis) versus the ratio of DNaseI-seq scores at stage 5 over those at 10 (y axis). (B) Plot of the ratio of ChIP-chip scores at stage 5 over those at 10 (x axis) versus the ratio of DNase-seq scores at stage 5 over those at 10 (y axis). (C) Plot of the ratio of ChIP-chip scores at stage 10 over those at 5 (x axis) versus the ratio of DNase-seq scores at stage 10 over those at 5 (y axis). (D) Plot of the ratio of ChIP-chip scores at stage 14 over those at 5 (x axis) versus the ratio of DNase-seq scores at stage 14 over those at 5 (y axis). The Pearson correlation coefficients (r) for each comparison are shown in the top right of each panel.

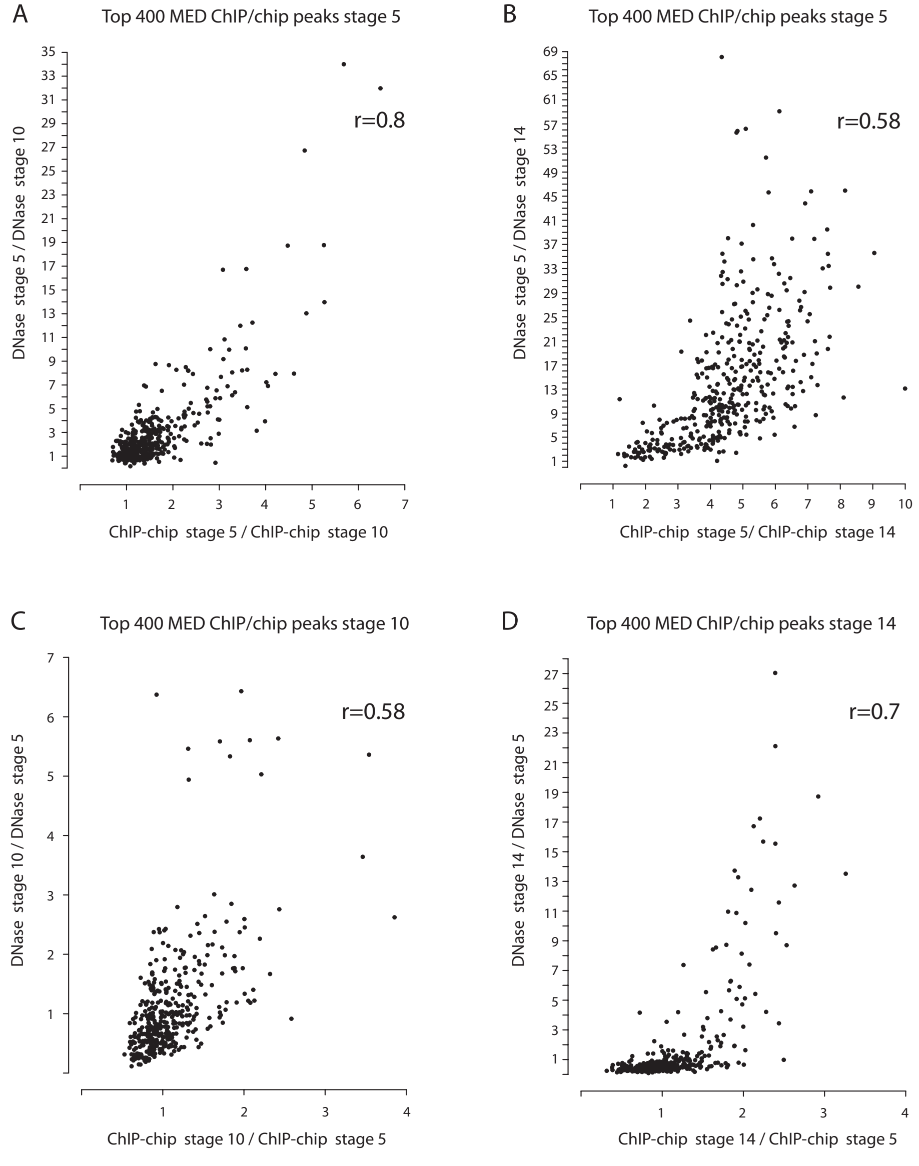

Supplement: Additional file 11 — Temporal changes in levels of MED occupancy correlate with changes in DNaseI accessibility. [file gb-2011-12-4-r34-S11.PDF]
